# Structural, functional and biological insights into the role of *Mycobacterium tuberculosis* VapBC11 toxin–antitoxin system: targeting a tRNase to tackle mycobacterial adaptation

**DOI:** 10.1093/nar/gky924

**Published:** 2018-10-17

**Authors:** Amar Deep, Prabhakar Tiwari, Sakshi Agarwal, Soni Kaundal, Saqib Kidwai, Ramandeep Singh, Krishan G Thakur

**Affiliations:** 1 Structural Biology Laboratory, G. N. Ramachandran Protein Centre, Council of Scientific and Industrial Research-Institute of Microbial Technology (CSIR-IMTECH), Chandigarh 160036, India; 2 Tuberculosis Research Laboratory, Vaccine and Infectious Disease Research Centre, Translational Health Science and Technology Institute, NCR-Biotech Science Cluster, 3^rd^ Milestone, Faridabad Gurgaon Expressway, Faridabad 121001, India

## Abstract

Toxin–antitoxin (TA) systems are involved in diverse physiological processes in prokaryotes, but their exact role in *Mycobacterium tuberculosis* (*Mtb*) virulence and *in vivo* stress adaptation has not been extensively studied. Here, we demonstrate that the VapBC11 TA module is essential for *Mtb* to establish infection in guinea pigs. RNA-sequencing revealed that overexpression of VapC11 toxin results in metabolic slowdown, suggesting that modulation of the growth rate is an essential strategy for *in vivo* survival. Interestingly, overexpression of VapC11 resulted in the upregulation of chromosomal TA genes, suggesting the existence of highly coordinated crosstalk among TA systems. In this study, we also present the crystal structure of the VapBC11 heterooctameric complex at 1.67 Å resolution. Binding kinetic studies suggest that the binding affinities of toxin–substrate and toxin–antitoxin interactions are comparable. We used a combination of structural studies, molecular docking, mutational analysis and *in vitro* ribonuclease assays to enhance our understanding of the mode of substrate recognition by the VapC11 toxin. Furthermore, we have also designed peptide-based inhibitors to target VapC11 ribonuclease activity. Taken together, we propose that the structure-guided design of inhibitors against *in vivo* essential ribonucleases might be a novel strategy to hasten clearance of intracellular *Mtb*.

## INTRODUCTION

Tuberculosis (TB), caused by *Mycobacterium tuberculosis* (*Mtb*), is a leading cause of mortality worldwide (1). The challenges in eradicating TB are rising due to the emergence of various drug-resistant strains, HIV co-infection and failure of the BCG vaccine to impart protection against adulthood pulmonary TB (2–4). *Mtb* is a highly successful intracellular pathogen because of its ability to exist in an altered metabolic state that is phenotypically drug-tolerant (5). Numerous studies have identified various metabolic pathways, such as protein kinases, stringent response, two-component systems, sigma factors and toxin–antitoxin (TA) modules, as the key determinants of bacterial virulence (6–10).

TA systems are widespread in prokaryotes and are involved in diverse physiological processes, such as stress adaptation, plasmid maintenance, phage protection, virulence and biofilm formation (11,12). TA systems exist as cognate pairs of either protein–protein or protein–RNA molecules (13). Based on the nature of antitoxins and the mechanism of toxicity neutralization, TA systems have been broadly classified into six types—Type I–VI (14). Thus far, the reported toxins belonging to TA systems are proteins harbouring attributes, such as DNase, RNase, enzyme modification and membrane potential disruption capabilities (12,13). The overexpression of Type II toxins inhibit bacterial growth in either a bactericidal or bacteriostatic manner (15,16). In Type II TA systems, both toxin and antitoxin form a tight TA complex that binds to the operator region and results in auto-repression (17). The N-terminal domain of antitoxins possess DNA-binding properties, and the C-terminal domain neutralizes the toxin (18,19). Several DNA-binding motifs, such as ribbon-helix-helix (RHH), helix-turn-helix, AbrB and PhD/YefM, have been mapped to the N-terminal domain of antitoxins (20,21). However, upon exposure to stress conditions, antitoxins are degraded by cellular proteases in a (p)ppGpp-dependent manner, and subsequently, the free toxins cleave their cellular targets (22).

Virulence-associated proteins B and C (VapBC) are the most abundant Type II TA system present in prokaryotes (23). VapC, the toxin, inhibits protein translation by cleaving either transfer RNA (tRNA) or the sarcin-ricin loop (SRL) of 23S ribosomal RNA (rRNA) or messenger RNA (mRNA), and this activity is neutralized by their cognate VapB antitoxins (24). The *Mtb* genome encodes for 90 TA systems, and the majority of these (50 TA modules) belong to the VapBC family (10,25). Interestingly, *Mycobacterium smegmatis* (*Msm*), a non-pathogenic mycobacterial species, harbours only a single VapBC module, suggesting their importance in *Mtb* pathogenesis (26). Several structures, including VapBC3, VapBC5, VapBC15, VapC20, VapC21, VapBC26 and VapBC30 (27–33), have been resolved, but there is very limited information about their role in bacterial physiology (10). Though these toxins share poor sequence identity (<30%), they all adopt characteristic PIN domain architecture (34). Recently, we have shown that *Mtb* TA systems are differentially expressed, regulated in a post-transcriptional manner and inhibited *Mtb* growth in a bacteriostatic manner (7,10,35). We have also shown that amongst several VapC toxins, the overexpression of VapC11 also inhibited growth of *Mtb* (10). VapC11 was amongst the few VapCs that were upregulated in response to exposure to various antibiotics, suggesting its crucial role in stress adaptation (10,25). This prompted us to perform a detailed structural and functional characterization of the VapBC11 TA system from *Mtb*.

In the present study, we show that the VapBC11 TA system is dispensable for *in vitro* growth but is essential for *Mtb* to establish disease *in vivo*. We observed that the overexpression of VapC11 resulted in major alterations in the transcriptional profiles of *Mtb*. These profiles were similar to the profiles of nutritionally starved bacteria, those enduring hypoxic response (EHR), non-replicating persisters (NRP) and drug-induced persisters (36–39). Furthermore, we solved the crystal structure of VapC11 in complex with its cognate antitoxin VapB11 at 1.67 Å resolution. Structure-guided inhibitor design led to the identification of antitoxin-based peptides that inhibited the VapC11-mediated cleavage of tRNA-Leu^CAG^ in a concentration-dependent manner. We also performed solution studies to determine the binding stoichiometry and the mechanism of toxin inhibition. Taken together, this study unravels the essentiality of VapBC11 in mycobacterial pathogenesis. The inhibition of the ribonuclease activity of VapC11 and/or other *in vivo* essential toxins might form a rationale to design inhibitors that possess activity against both drug-susceptible and drug-resistant bacteria.

## MATERIALS AND METHODS

### Construction of knockout and overexpression strains

The *ΔvapBC11* mutant strain was generated using temperature-sensitive mycobacteriophage phAE87, as described previously (40). The replacement of *vapBC11* locus with the hygromycin resistance gene was confirmed by polymerase chain reaction (PCR) and Southern blot analysis. The construction of the overexpression strains was carried out as previously described (10). A list of all the primers used in this study is provided in Supplementary Table S1.

### Bacterial viability studies

The effect of VapC11 overexpression on *M. bovis* BCG viability was studied using BacLight LIVE/DEAD bacterial viability kit, as per manufacturer’s recommendations (Life Technologies). Briefly, single-cell suspensions were prepared and stained with SYTO-9/propidium iodide mix. The stained bacilli were viewed and imaged using a confocal microscope (FV1000 Olympus) with Fluoview software (Olympus Tokyo, Japan).

### RNA-Sequencing and data analysis

For RNA-Seq analysis, total RNA was isolated from VapC11 overexpressing strains and control cells at 24 h post-anhydrotetracycline addition. Total RNA was purified and shipped to Aggrigenome Labs Pvt. Ltd. (India). RNA integrity was checked using the Agilent RNA 6000 Nano Kit (Agilent Technologies, Santa Clara, CA, USA). rRNA was removed from the total RNA sample using a Ribo-Zero™ Magnetic kit (Bacteria), as per manufacturer’s recommendations (Epicentre). RNA sequencing was performed according to protocols standardized in Aggrigenome Labs Pvt. Ltd. The reads that passed quality control were mapped onto the *Mtb* reference genome using TopHat2 (41). The abundance of transcripts in individual samples was estimated using Cufflinks (42). Differential gene expression was assessed in VapC11-overexpressing samples with respect to control samples using Cuffdiff. Genes showing expression levels ≥log2-fold change in either direction with a *P*-value <0.05 were considered for downstream analysis. The DAVID functional annotation tool was used for gene set enrichment analysis of the differentially expressed genes (DEGs) (43). For data visualization and presentation, the R software package was used.

To validate the identified DEGs in VapC11 overexpression strains, quantitative PCR (qPCR) experiments were performed. Complementary DNA synthesis and qPCR analysis using gene-specific primers was performed as previously described (35). The expression of genes of interest was normalized to the transcript levels of *sigA* (a housekeeping gene) and was quantified as previously described (35).

### *In vitro* stress experiments

*Mtb* wild-type, *ΔvapBC11* and *vapBC11* CT strains were cultured in MB 7H9 (Middlebrook 7H9) medium, and their growth pattern was observed by measuring the absorbance at 600 nm. For *in vitro* stress experiments, early logarithmic cultures of different strains were exposed to either oxidative stress (5 mM H_2_O_2_) for 24 h or nitrosative stress (5 mM NaNO_2_) for 72 h or nutritional stress (1× Tris-buffered saline including Tween-80) for 7 days, as previously described (6). For *in vitro* drug tolerance, mid-log phase cultures were subsequently exposed to drugs with different mechanisms of action, including rifampicin (transcription inhibitor), levofloxacin (replication inhibitor) and isoniazid (cell wall inhibitor), as previously described (35). Macrophage infection experiments were performed at an multiplicity of infection (MOI) of 1:10 as previously described, and colony forming units (CFU) enumeration was performed at day 0, 2, 4 and 6 post infection (10). For CFU enumeration, 100 μl of 10.0 serial dilutions were plated on MB 7H11 medium at 37°C for 3–4 weeks.

### *In vivo* guinea pig experiments

The animal experiments were conducted with prior approval from the Institutional Animal Ethics Committee of the International Centre for Genetic Engineering and Biotechnology and Translational Health Science and Technology Institute. The animal experiments were performed in agreement with the guidelines provided by Committee for the Purpose of Control and Supervision of Experiments on Animals (CPCSEA, Government of India). Female guinea pigs (Hartley strain, 200–300 g) were procured from Lala Lajpat Rai University, Hisar and infected with single-cell suspensions of log-phase growing *Mtb* (10^8^) cultures via the aerosol route. CFU enumeration in different organs and histopathology analysis of haematoxylin and eosin (H&E) sections at designated time points were performed, as previously described (6).

### Protein purification

The DNA fragments coding for either VapC11 or VapC11^D5A^ were cloned into the multiple cloning site-I of pETDuet-N, as described previously for VapC20 (30). Consistent with previously published reports, we were unable to purify full-length VapB11 in its soluble form (30,44). However, we were able to express and purify a shorter VapB11 form (residues 6–72), and we refer to this shorter version of VapB11^6–72^ as VapB11. Briefly, *vapB11* was cloned into the pNIC28-Bsa4 vector to yield (6×-His)-VapB11 protein. *vapC11* and various single mutants were cloned into arabinose-inducible modified pBAD-Myc-HIS vector (30). All constructs generated in the study were verified by DNA sequencing. For the co-expression and co-purification of the VapBC11 TA complex, *vapB11* was cloned into the MCS II of the pET-Duet-N-*vapC11* and pETDuet-N-*vapC11^D5A^* constructs. Construction of VapBC11^D5A^ complex allowed us to purify this complex with relatively higher yield, which was required in the initial rounds of crystallization trials.

For protein purification, all of the constructs were transformed into *Escherichia coli* (*E. coli*.) strain Rosetta DE3 (Novagen). When the OD_600_ value reached ∼0.7, protein expression in the transformants was induced by the addition of 0.3 mM isopropyl β-D-1-thiogalactopyranoside, with incubation for 16 h at 16°C and constant shaking. The induced cultures were harvested by centrifugation at 7000 g for 10 min at 4°C. For pBAD constructs, the expression of protein was induced with the addition of 0.2% L-arabinose at 37°C for 2 h, followed by harvesting. The purification of His-tagged proteins from clarified lysates was performed using Ni-NTA resin (Gold Biotechnology), as per the manufacturer’s protocol. Protein identity was confirmed using peptide mass fingerprinting. MALDI-TOF experiments were performed using AB-SCIEX. The purified active toxin and other variants were further purified by gel filtration chromatography using Superdex™ 200 Increase 10/300 GL (GE Healthcare) resin in a buffer containing 20 mM 4-(2-hydroxyethyl)-1-piperazineethanesulfonic acid (HEPES) (pH 8.0) and 150 mM NaCl.

### VapBC11 complex crystallization and structure solution

The purified VapBC11 complex was subjected to crystallization trials using commercially available screens from Hampton Research, USA and Molecular Dimensions, UK. High-throughput 96-well sitting-drop vapour diffusion crystallization trays were set up using an NT8 robotics system (Formulatrix Inc., USA). The drops contained 150 nl of protein sample and 150 nl of reservoir solution. The crystallization trays were incubated at 20°C in a Rock Imager 1000 (Formulatrix) for storage and automatic prescheduled imaging. The VapBC11^D5A^ complex (10 mg/ml protein concentration) crystallized in a condition containing 0.1 M ammonium sulphate, 0.3 M sodium formate, 0.1 M sodium cacodylate, 3% w/v PGA-LM and 30% polyethylene glycol (PEG) 400 (pH 6.5). Initial crystals appeared after 3 days of incubation and diffracted up to 6 Å. Further optimization using an oil microbatch-under-oil crystallization method resulted in better diffraction quality crystals of VapBC11. The drops were set up manually using 1.5 μl of protein and 1.5 μl of the crystallization conditions. The crystals appeared after 2 weeks of storage at 18°C and diffracted up to 1.67 Å at the ID29 beamline, ESRF, France. The data were processed using iMOSFLM and scaled using SCALA (45,46). The structure was solved by the molecular replacement method using PHASER (47). The *Mtb* VapBC15 (PDB ID: 4CHG) structure was used as a search model (29). Iterative steps of manual model building using COOT and refinement using Phenix. Refine resulted in a structure with a final *R*_work_/*R*_free_ of 0.183/0.209 (48,49). Crystal structure analysis was carried out using PYMOL and COOT (48,50). Structural homologues were identified using the DALI server (51). The oligomeric states and interface interactions in VapB11 and VapC11 were identified using the PDBePISA online server at http://www.ebi.ac.uk/pdbe/pisa/ (52).

### Analytical ultracentrifugation (AUC)

Analytical ultracentrifugation (AUC) experiments were performed using a Beckman-Coulter XL-A analytical ultracentrifuge with a TiAn50 eight-hole rotor. The sedimentation velocity experiments were performed at 40 000 rpm at 25°C. Two-channel epon centrepieces and quartz windows were used. Protein concentrations of 10, 20 and 30 μM were used in these experiments. The AUC experiments for VapB11, VapC11 and VapBC11 were performed in 20 mM HEPES (pH 8.0) and 150 mM NaCl. The absorbance scans were performed at 280 nm wavelength, and data scans were collected at 3 min intervals. The data obtained were fitted using the continuous distribution c(s) model in SEDFIT (53). The solvent viscosity and solvent density at 25°C were calculated using SEDNTERP (54).

### Surface plasmon resonance (SPR)

Surface plasmon resonance (SPR) experiments were performed to measure the binding kinetics of the VapC11 and VapB11 interactions using a Biacore 3000 (GE Healthcare). VapC11 toxin was resuspended at a concentration of 2 μM in 10 mM sodium acetate (pH 5.5) and immobilized on a CM5 sensor chip at up to 750 response unit. Running buffer (10 mM HEPES (pH 7.5) and 150 mM NaCl) was sterilized using a 0.2 μm filter. The experiments were performed at 25°C, with a continuous buffer flow rate of 20 μl/min. For kinetic studies, association and dissociation were observed for 300 and 500 s, respectively. Five different increasing concentrations of VapB11 were injected, and each experiment was repeated at least twice. The surface of the CM5 chip was regenerated by injecting multiple pulses (3–5) of 5 μl of 10 mM NaOH at a flow rate of 5 μl/min. The obtained sensorgrams were analysed using BIA evaluation software 3.0 with a 1:1 Langmuir binding model and drifting baseline.

### *In vitro* transcription, tRNA cleavage and peptide inhibition assays

For *in vitro* ribonuclease assays, *leuT* (tRNA-Leu^CAG^), along with its 150 bp upstream and downstream region (to avoid non-specific amplification, as tRNA genes are conserved at their ends), was cloned into the pET-Duet vector. The *in vitro* transcription reaction was performed to synthesize tRNA using T7 RNA polymerase (New England Biolabs). PCR amplification was performed using the *leuT*-specific forward primer, containing an overhanging T7 promoter sequence, and reverse primers using pETDuet-N-*leuT* as a template. The amplified product was purified using a GeneJET Gel Extraction kit (Thermo Scientific). For *in vitro* transcription assays, ∼200 ng of purified DNA was used as a template in a final reaction volume of 40 μl, as per the manufacturer’s protocol (New England Biolabs). The transcribed product was purified using phenol:chloroform extraction, followed by sodium acetate precipitation. The precipitated tRNA was washed twice with ice-cold ethanol, air dried and resuspended in nuclease-free water. The tRNA samples were refolded using a published protocol (55). Briefly, the tRNA samples were heated to 95°C and allowed to cool to 65°C in a thermomixer (Eppendorf). After adding MgCl_2_ to a final concentration of 10 mM, the samples were allowed to cool slowly to reach 25°C. The ribonuclease assays, using transcribed tRNA, were performed in cleavage buffer (10 mM HEPES (pH 8.0), 15 mM KCl, 1 mM DTT and 10 μM MnCl_2_) containing 10 μM of protein at 37°C for the mentioned time points. The reaction was stopped by the addition of formamide RNA loading dye and heating the sample at 70°C for 5 min. The samples were resolved on 15% urea-polyacrylamide gel electrophoresis gels and visualized by EtBr staining.

### Bio-layer interferometry (BLI)

To study the interactions of VapC11^D5A^ with tRNA-Leu^CAG^, we used ForteBio Octet RED 96 (ForteBio, USA) and CM5 sensors (ForteBio, USA). The experiment was conducted at 25 °C in a buffer containing 20 mM HEPES (pH 7.5) and 150 mM NaCl. VapC11^D5A^ diluted in 10 mM sodium acetate buffer (pH 5.0) was immobilized using EDC/NHS chemistry on CM5 sensor tips, as per the manufacturer’s recommendations. Before use, the sensor tips were hydrated in assay buffer for 20  min. A 200  μl aliquot of either sample or buffer was added to a 96-microwell plate with 1000 rpm rotation. VapC11^D5A^ at a concentration of 1 nM was immobilized onto the working sensor tip. Another reference sensor without immobilized protein was subjected to the identical procedure for double referencing and to subtract non-specific tRNA binding with the sensor material. Following immobilization with VapC11^D5A^, the binding interaction with different concentrations (25, 100 and 200 nM) of tRNA was performed. Briefly, after a stable baseline was observed, association (350 s) and dissociation (350 s) were monitored, followed by cyclic regeneration/neutralization with 5 mM NaOH and assay buffer. The obtained data were analysed, and the*K*_D_ for substrate binding was calculated by the curve fit (1:1) model using ForteBio data analysis 9.0 software.

## RESULTS

### Ectopic expression of VapC11 results in bacteriostasis and alteration in global transcriptional profile

**Figure 1.**
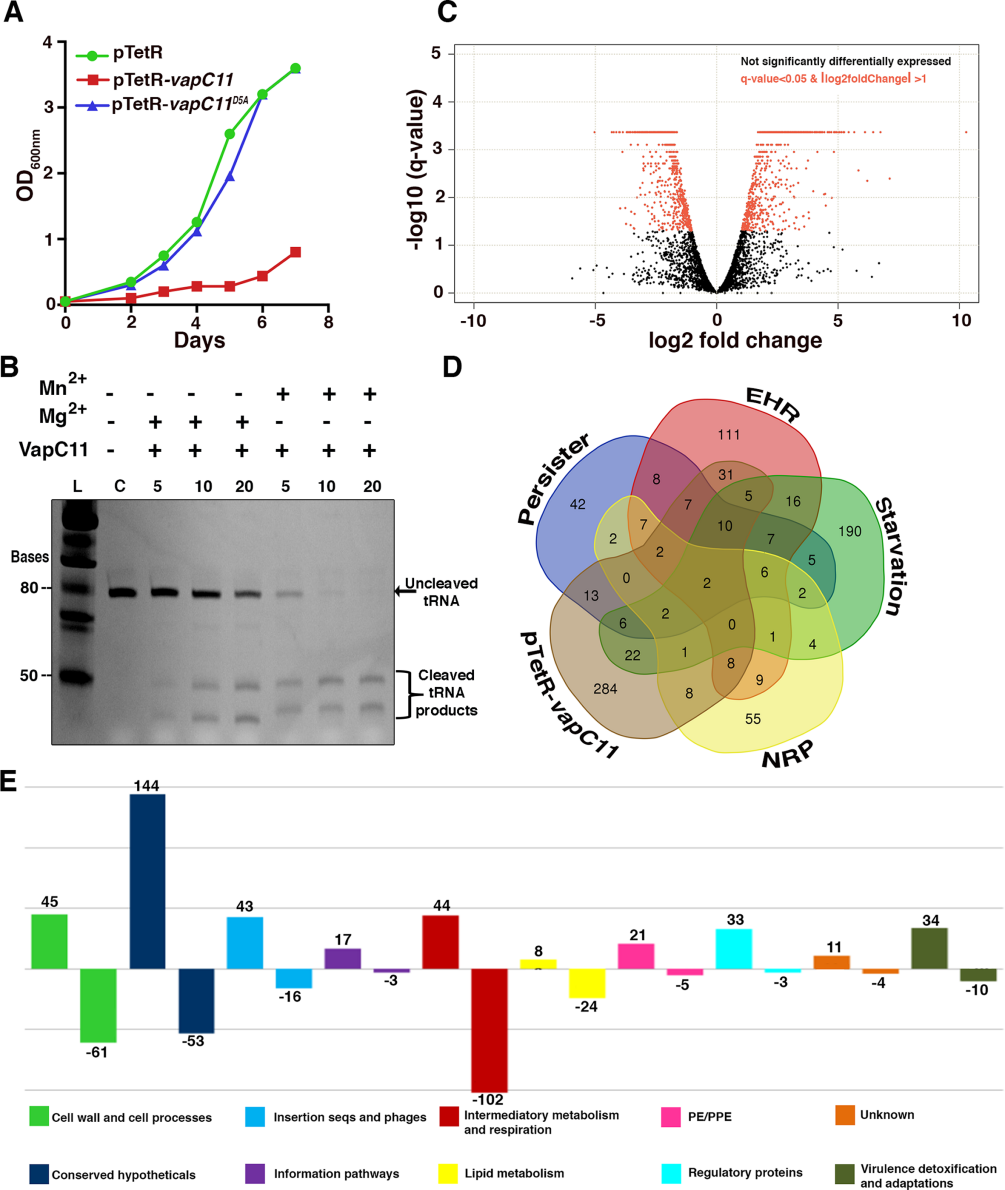
Overexpression of VapC11 induces bacteriostasis and large-scale changes in the *Mtb* transcriptome. (**A**) The expression of VapC11 and VapC11^D5A^ in *Mtb* was induced by the addition of 50 ng/ml anhydrotetracycline. The growth of various strains was determined by measuring absorbance at 600 nm. (**B**) *In vitro* VapC11 tRNA cleavage assay in the presence of Mg^2+^ and Mn^2+^ metal ion(s). The retarded mobility observed in the cleaved RNA bands is probably due to the presence of metal ion(s) in the reactions. (**C**) Volcano plot displaying the gene expression profile from 24 h post VapC11-induced samples. The *Y*-axis depicts *q*-values for each gene and the *X*-axis depicts fold change in either direction. (**D**) Venn diagram showing the correlation of differentially upregulated genes in VapC11 overexpression strain (pTetR-*vapC11*) versus nutrient starved (starvation), persister, NRP and EHR transcriptome. (**E**) Bar plot showing the number of DEGs with their pathway category/involvement, as annotated in the Mycobrowser, https://mycobrowser.epfl.ch/.

VapC toxins inhibit protein translation by cleaving either the tRNA, 23S rRNA or mRNA (24,44,56,57). Previously, we have shown that VapC11 is differentially expressed in different stress conditions and inhibits bacterial growth in a bacteriostatic manner (10,25). As expected, mutating the first aspartic acid residue (Asp5) of the PIN domain abrogated the toxicity associated with VapC11. These results reaffirmed that the observed growth inhibition was due to the ribonuclease activity associated with VapC11 (Figure 1A). In accordance with previous reports, we observed that *M. bovis* BCG overexpressing VapC11 were viable using the BacLight LIVE/DEAD bacterial viability kit (Supplementary Figure S1A). Recently, it was reported that VapC11 inhibits protein translation by cleaving *Mtb* tRNA-Leu^CAG^ (24). In the present study, we also show that VapC11 cleaves tRNA-Leu^CAG^ in a Mg^2+^-dependent manner (Figure 1B). The crystal structure of VapBC15 has been shown to contain an additional Mn^2+^ metal ion adjacent to the Mg^2+^-bound active site (29). In the present study, we also examined the role of Mn^2+^ ions in the ribonuclease activity of VapC11 using tRNA-Leu^CAG^ as a substrate. Interestingly, the addition of 10 μM Mn^2+^ ions to the assay buffer significantly enhanced VapC11 tRNase activity by ∼5-fold (Figure 1B). Previous reports have also suggested that Mn^2+^ ions play an important role in VapC ribonuclease activities (29,32); however, none of these studies tested ribonuclease activity in the presence of natural tRNA substrates. To the best of our knowledge, this is the first report suggesting the role of physiological Mn^2+^ concentrations (∼20 μM in *E. coli* (58)) in enhancing the activity of a VapC toxin.

Next, we investigated the effect of VapC11 overexpression on the *Mtb* transcriptome. The data obtained suggest that the ectopic expression of VapC11 results in significant changes in the transcriptional profiles. As shown in Figure 1C and E, overexpression of VapC11 led to the upregulation and downregulation (≥log 2.0-fold) of 400 and 281 genes, respectively. The list of DEGs in VapC11 overexpression strains is summarized in Supplementary Table S2. The expression patterns for some of the DEGs identified in the transcriptomic analysis were further validated by qPCR using gene-specific primers (Supplementary Figure S1B). We next performed a detailed comparative analysis of the DEGs obtained using previously published transcriptome profiles that were observed in bacteria exposed to different stress conditions. As shown in Figure 1D, the profiles obtained were strikingly similar to those reported for nutritionally starved bacteria, those undergoing the EHR, NRPs and drug-induced persisters (36–39). These findings suggest that the presence of free toxin results in a significant reduction of *Mtb* metabolism. Interestingly, both Rv3290c and Rv2660c were upregulated in all datasets, suggesting their pivotal roles in stress adaptation. Rv3290c (L-lysine-epsilon aminotransferase or *lat*) is shown to be involved in persister formation via modification of the intracellular amino acid levels (59). Rv2660c is a hypothetical gene known to be upregulated in several stress conditions (39). An Rv2660c-overexpressing strain is being evaluated as a potential vaccine candidate (60). Five genes, Rv1128c, Rv1144, Rv1405, Rv1831 and Rv2407, which are essential for the survival of *Mtb* in both macrophages and mice models, were also upregulated in our study (61,62). We observed that the downregulated DEGs included several genes involved in the metabolic pathways required for either respiration or glycolysis or ATP/NADPH synthesis and protein translation (Supplementary Table S2).

We had previously reported that TA systems are activated in a post-transcriptional manner (10). In accordance with this, 23 chromosomal toxin or antitoxin genes were upregulated in VapC11-overexpression strains, thereby implying that a highly coordinated crosstalk exists among TA systems (Supplementary Table S2). Interestingly, 7 out of 10 TA pairs that were reported to be upregulated in the EHR, nutrient starvation conditions, and drug-induced persisters were also induced in VapC11-overexpressing strains (36–39). These TA genes include Rv2034/Rv2035, Rv3188/Rv3189, *higA*, Rv3181c/Rv3180c, Rv0918, Rv1990c/Rv1989c, Rv2021/2022, *vapB3* and *vapBC31*. These findings imply that a common mechanism for TA activation exists in *Mtb* upon exposure to different stress conditions. Similarly, other stress-responsive genes, such as *clpC2*,*sigE*,*PE/PPE* pathway genes and several hypothetical genes, were also upregulated in our study (Supplementary Table S2). Taken together, these results suggest that the presence of free VapC11 induces a metabolic slowdown and consequently growth inhibition, which possibly enables *Mtb* to adapt to different stress conditions in the host.

### VapBC11 is essential for *Mtb* to establish *in vivo* infection

Previously, we had reported that VapBC TA systems are differentially expressed and are also regulated in a post-transcriptional manner (10). Both MazF and VapC ribonucleases have been shown to be essential for establishing infection in guinea pigs (7,10). Here, we evaluated the contribution of the VapBC11 TA system in *Mtb* physiology and virulence. We generated a *vapBC11* knockout strain (*ΔvapBC11)* of *Mtb* using temperature-sensitive mycobacteriophages (Supplementary Figure S2A). The replacement of the *vapBC11* locus with the hygromycin resistance gene (*hyg*) in the *Mtb* genome was confirmed by both PCR and Southern blot (Supplementary Figure S2B and S2C). For the construction of the complemented strain, pMV306K-*vapBC11* was electroporated in the *ΔvapBC11* strain. The restoration of expression of the *vapBC11* locus in the complemented strain (*vapBC11 CT*) was confirmed by qPCR using gene-specific primers. The colony morphology and growth patterns of *ΔvapBC11* in liquid cultures were similar to those seen for the parental strain. We observed that the mutant strain had a statistically significant growth defect (∼2.0-fold) upon exposure to oxidative stress conditions in comparison to the parental strain (Supplementary Figure S3A, *P < 0.05). In accordance with our earlier observations, in the case of *ΔvapBC3, ΔvapBC4* and *ΔvapC28*, we observed that the VapBC11 TA locus was dispensable for *Mtb* survival upon exposure to other tested *in vitro* stress conditions, such as nitrosative, hypoxia, nutritional, NRP, macrophage and antimycobacterial drugs (Supplementary Figure S3B–F).

**Figure 2.**
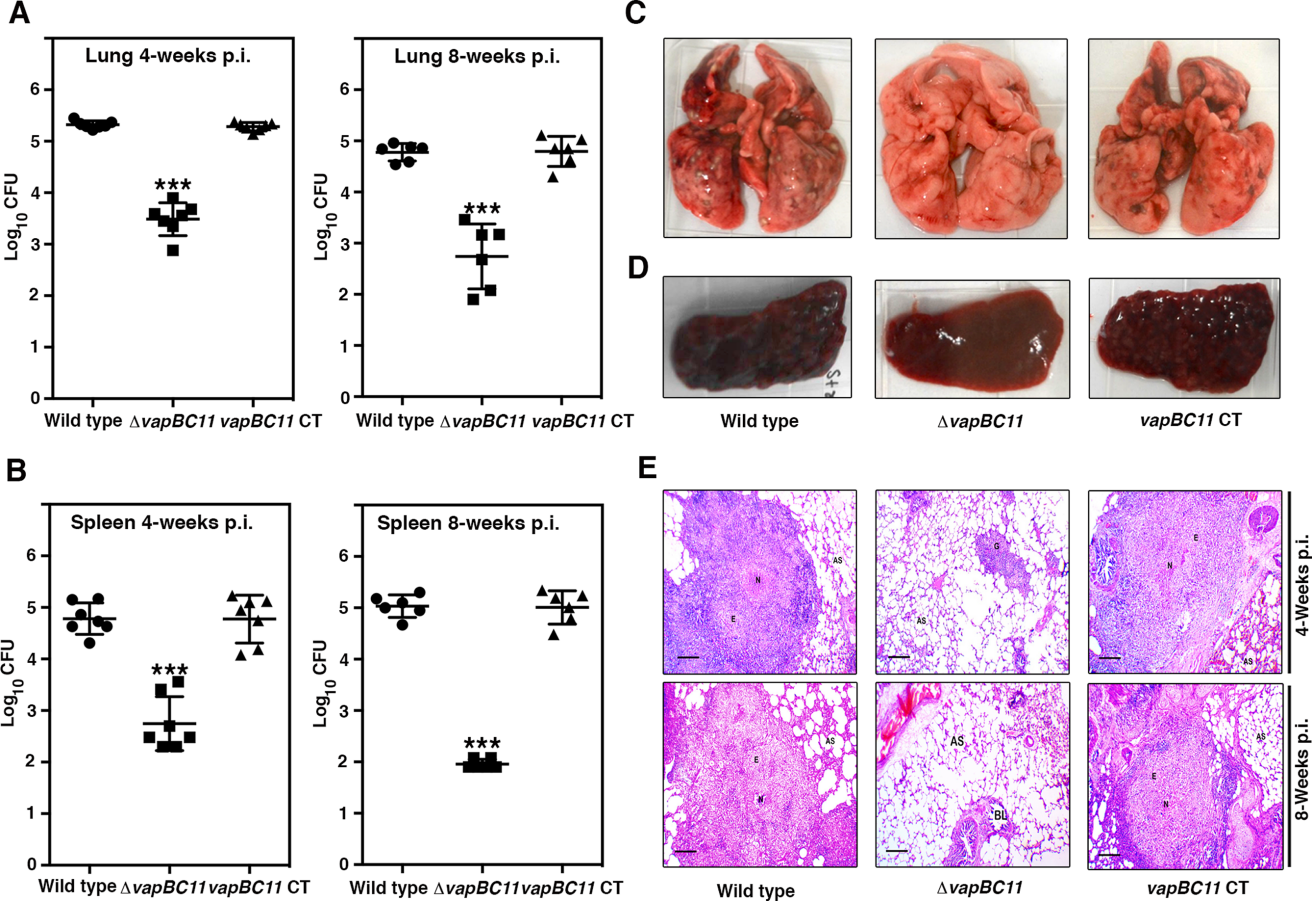
Effect of *vapBC11* deletion on mycobacterial virulence. (**A** and **B**) Guinea pigs (Dunkin–Hartley strain) were infected with *Mtb H37Rv, ΔvapBC11 and vapBC11* CT strains via the aerosol route. Panels showing bacterial loads in lungs and spleens of infected guinea pigs at either 4 or 8 weeks post infection. Each data point represents the log_10_ CFU from an individual animal. The data plotted here are the mean ± S.E. calculated for each group. Significant differences were observed for the indicated groups (paired [two-tailed] *t*-test, *** represents *P*-value <0.001). (**C** and **D**) Panels showing representative lungs and spleen images from each animal group at 8 weeks post infection. (**E**) Panels depicting the histopathology photomicrographs of samples from lung sections at 4 and 8 weeks post-infection time points. Samples were stained using haematoxylin and eosin dyes. Abbreviations: AS, alveolar space; BL, bronchial lumen; E, epithelial cells; G, granuloma; N, necrosis. Scale bar size: 100 and 200 μM for 4 and 8 weeks post-infection time points.

To investigate the contribution of the VapBC11 TA system to the ability of *Mtb* to establish infection in the host, guinea pigs were infected with various strains via the aerosol route. In comparison to the parental strain, lung bacillary loads of *ΔvapBC11*-infected guinea pigs were reduced by 80- and 100-fold at 4 and 8 weeks post infection, respectively (Figure 2A, ***P < 0.001). As shown in Figure 2B, a statistically significant 100-fold reduction in splenic bacillary loads was observed in mutant strain-infected guinea pigs (***P < 0.001). This difference in splenic bacillary load further increased to 1000-fold 8 weeks post infection (Figure 2B, ***P < 0.001). This growth defect associated with the mutant strain was restored in guinea pigs infected with the *vapBC11 CT* strain. As shown in Figure 2A and B, the growth patterns of the complemented strain were similar to those observed in guinea pigs infected with the parental strain. In agreement with the bacterial loads, less tissue damage was seen in the lungs and spleens of guinea pigs that were infected with the mutant strain, in comparison to the animals infected with either the parental or complemented strain (Figure 2C and D). Similarly, we observed significantly reduced cellular infiltration and granuloma formation in H&E-stained sections from *ΔvapBC11*-infected guinea pigs compared to the sections from guinea pigs infected with the parental and *vapBC11 CT* strains (Figure 2E). Taken together, these results demonstrate the essentiality of VapBC11 TA systems for *Mtb* to establish infection in guinea pigs.

### Crystal structure of the VapBC11 complex

**Figure 3.**
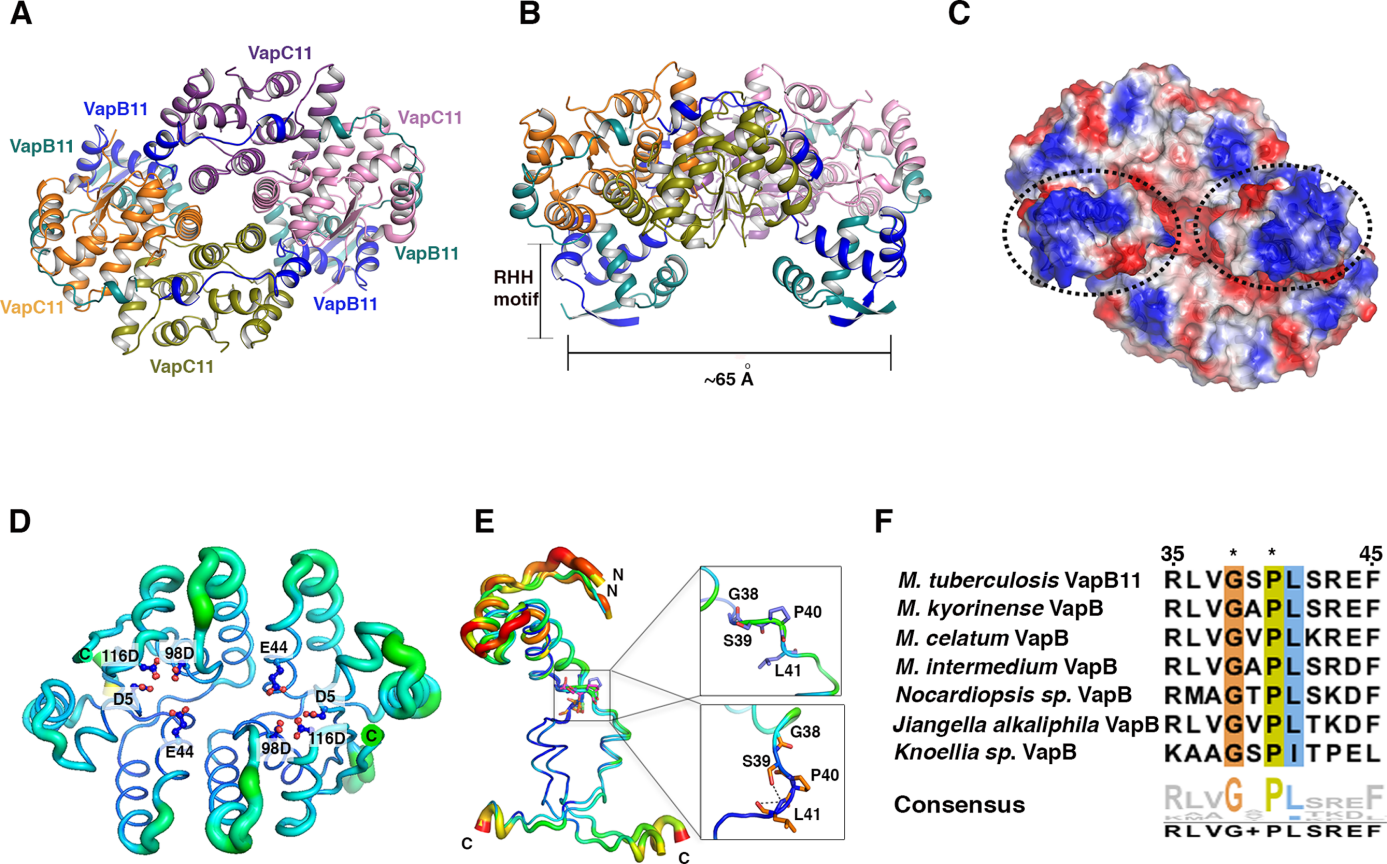
Crystal structure of VapBC11 complex. (**A** and **B**) Ribbon representations of VapBC11 TA complex in two different orientations. Each chain is shown in a different colour and labelled with the respective colour. RHH is a ribbon-helix-helix DNA-binding motif at the N-terminal region of VapB11. (**C**) Electrostatic surface potential charge distribution of VapBC11 heterooctameric complex. Red and blue colours indicate negative and positive charge, respectively. Hydrophobic patches are shown in white colour. The dotted circles represent positively charged DNA-binding domains. (**D**) The B-factor putty representation of VapC11 toxin structures. Two VapC11 homodimers are superimposed on each other. The thickness of the tube represents thermal vibration or flexibility. The residues involved in the interactions are shown in boxes. (**E**) The B-factor putty representation of VapB11 antitoxin chains. All four VapB11 chains are superimposed on each other. N- and C-terminal domains are linked with a flexible linker (Gly-X-Pro) that allows VapB11 to adopt two distinct conformations. Residues preferred at specified positions for domain flexibility are shown in square boxes with stick presentation. (**F**) Multiple sequence alignment of VapB homologues showing the conservation of Gly-X-Pro residues marked as asterisks.

The essentiality of VapBC11 to establish infection *in vivo* suggests that this TA module can be exploited as a potential drug target. To this end, we determined the crystal structure of VapC11 in complex with its cognate antitoxin VapB11. The VapBC11 complex crystallized in the P2_1_2_1_2_1_ space group, and the structure was solved at 1.67 Å resolution using molecular replacement, as described in the ‘Materials and Methods’ section. The asymmetric unit consisted of four chains each of VapB11 and VapC11 (Figure 3). The data collection and refinement statistics are summarized in Table 1. There were no residues in the disallowed region of the Ramachandran plot in the final model (63). The final model includes residues Ser2-Ser63 of VapB11 (67 amino acids) and Ala2-Arg134 of VapC11 (134 amino acids) in all the chains (Figure 3). VapB11 and VapC11 interact to form a VapB_4_C_4_11 heterooctameric complex (Figure 3A and B). Both VapB11 and VapC11 appear to self-associate to form homodimers. The electrostatic potential distribution shows that the VapBC11 structure possesses both positively and negatively charged patches on its surface (Figure 3C). As expected, the active site of VapC11 is highly negatively charged, and the intertwined N-terminal RHH DNA-binding motif of VapB11 has a net positive charge (Figure 3C). The positive charge of this latter motif potentially contributes to the binding of the VapBC11 complex to its operator region. Superimposing the VapBC11 complex structure with the FitAB–DNA complex structure revealed that the distance between two RHH motifs of VapB11 is comparable to that observed for FitA antitoxins, which bind to major grooves of the *cis*-DNA element (Supplementary Figure S4) (64). As expected, VapC11 adopts canonical PIN domain fold architecture β(1)α(6)β(1)α(1)β(1) topology (Figure 3D). Similar to the other known VapC structures, the highly conserved PIN domain residues constitute an active site (Figure 3D and Supplementary Figure S5). The close structural homologues of VapC11 were identified using DALI server (51). *Mtb* VapC15 (PDB ID: 4CHG) (29) is the closest structural homologue, sharing 30% sequence identity and a root mean square deviation (rmsd) of 1.30 Å over 133 equivalent Cα positions. *Mtb* VapC21 (PDB ID: 5SV2) (31) shares 27% sequence identity with VapC11 and an rmsd of 1.94 Å over 124 equivalent Cα positions. There were several observable changes in the length and positioning of α-3 and α-4 helices with varying lengths of connecting loop regions (Supplementary Figure S6). These changes are crucial, as they are positioned in the vicinity of the active site and might facilitate substrate recognition and subsequent catalysis. Although the overall structure of VapC11 closely resembles the other known VapC structures, there are subtle differences in the C-terminal regions. The C-terminal region is structurally variable and adopts different conformations, such as α-helix, β-strand or disordered structure (33). Despite similarities in the tertiary structures, we observed subtle differences in the relative orientations of the monomers in the homodimers when VapC11 was superimposed on its close structural homologues VapC15 and VapC21 (Supplementary Figure S6A). These differences were primarily due to variations in the residues lining the dimeric interface, resulting in differences in the active site pocket architecture (Supplementary Figure S6A). Furthermore, differences in the overall electrostatic potential charge distribution were also observed, which, along with the overall shape of the active site pocket, likely plays a crucial role in determining the target specificity (Supplementary Figure S6B).

**Table 1.** Data collection and refinement statistics

	VapBC11
**Data collection**	
Space group	P 2_1_ 2_1_ 2_1_
Cell dimensions	
*a, b, c* (Å)	50.69, 127.61, 151.95
*α, β, γ* (°)	90, 90, 90
Resolution (Å)	48.63-1.67 (1.76-1.67)**
*R*_merge_	0.044 (0.881)
*I*/Σ*i*	15.7 (1.4)
CC1/2	0.999 (0.643)
CC*	0.999 (0.951)
Completeness (%)	99.7 (98.8)
Redundancy	4.6 (4.4)
Wavelength (Å)	1.072
No. of unique reflections	113 379 (16 176)
*R*_work_ /*R*_free_	0.183/0.209
Atom count	
Protein	6137
Ligand	26
Water	577
B-factor	
Protein	39.45
Ligand	52.30
Water	44.17
r.m.s.d.^#^	
Bond lengths (Å)	0.009
Bond angles (°)	1.30
Ramachandran plot statistics	
Most favoured (%)	96.79
Allowed regions (%)	3.21
Disallowed region (%)	0

**Values in parentheses correspond to the highest resolution shell.

^#^r.m.s.d., root mean square deviation.

Both N- and C-terminal regions of VapB11 were resolved well in the electron density, while in the crystal structure of the closest structural homologue, VapBC15 (PDB ID: 4CHG) (29), the N-terminal region could not be resolved due to disorder or degradation. The N-terminal domain (∼1–40 amino acids) in VapB11 forms a RHH structural motif (Figure 3B). The C-terminal region (residue range 41–63) and the α3 and α4 helices that are linked by an eight residue-long linker interact extensively with VapC11 (Figure 3A and B). We observed that the α3 helix docks at the lateral site, while α4 interacts with the active site residues of VapC11. Consequently, this mode of interaction may sterically occlude the tRNA-binding site of the toxin (Figure 3B). Therefore, each toxin molecule is neutralized by the binding of the cognate antitoxin molecule in the VapBC11 complex. The N-terminal region of VapB11 self-associates to form a RHH DNA-binding domain, and the C-terminal regions of the VapB11 homodimer crosslink VapC monomers of different homodimers (Supplementary Figure S7A). The α-helices of the VapB antitoxins that interact with VapC toxin in the lateral site vary in their lengths (Supplementary Figure S7B). The C-terminal residues of antitoxins, which mediates interactions with the active site residues, are either unstructured (PDB ID: 5K8J, 2BSQ) (64,65) or form small helices (PDB ID: 3H87, 4CHG, 4XGQ, VapB11) (Supplementary Figure S7A and S7B) (27,29). Though in the majority of the cases, VapB interacts with VapC in a 1:1 stoichiometry, interesting insights have been observed in a few structure where an extended C-terminus region interacts with the two active sites in the VapC dimers (5K8J and 4CHG) (Supplementary Figure S7A). In VapBC11, the interactions of VapB11 and VapC11 result in the formation of a heterooctamer. The overall architecture of VapBC11 adopts a closed conformation, where two VapC11 homodimers additionally interact at the α4 and α5 of diagonally positioned VapC chains (Figure 3A). In contrast, the heterooctameric complexes of other VapBC TA systems adopt the open conformation (66,67).

The presence of two independent VapC11 homodimers in an asymmetric unit allowed us to analyse detailed structural variations in the toxin and antitoxin using structural superposition and B-factor analysis. As expected, the loop regions are flexible, while the core regions including the active site region and the regions involved in binding with VapB11 are stable (Figure 3D). The N-terminal RHH domain in VapB11 is linked with a flexible linker region, which might facilitate its interactions with the *cis*-DNA element (Figure 3E). Superimposition of the four VapB11 RHH motifs from the crystal structure suggested that the C-terminal region adopts two distinct conformations facilitated by flexible Gly38-Ser39-Pro40 residues (Figure 3E). Sequence analysis revealed that a Gly-X-Pro sequence motif is conserved in closely related VapB homologues (Figure 3F). We speculate that the presence of this motif might provide the desired flexibility required for crosslinking VapC homodimers to form heterooctameric TA assembly (Figure 3F).

### VapB11 and VapC11 interact to form a heterooctameric complex in solution

**Figure 4.**
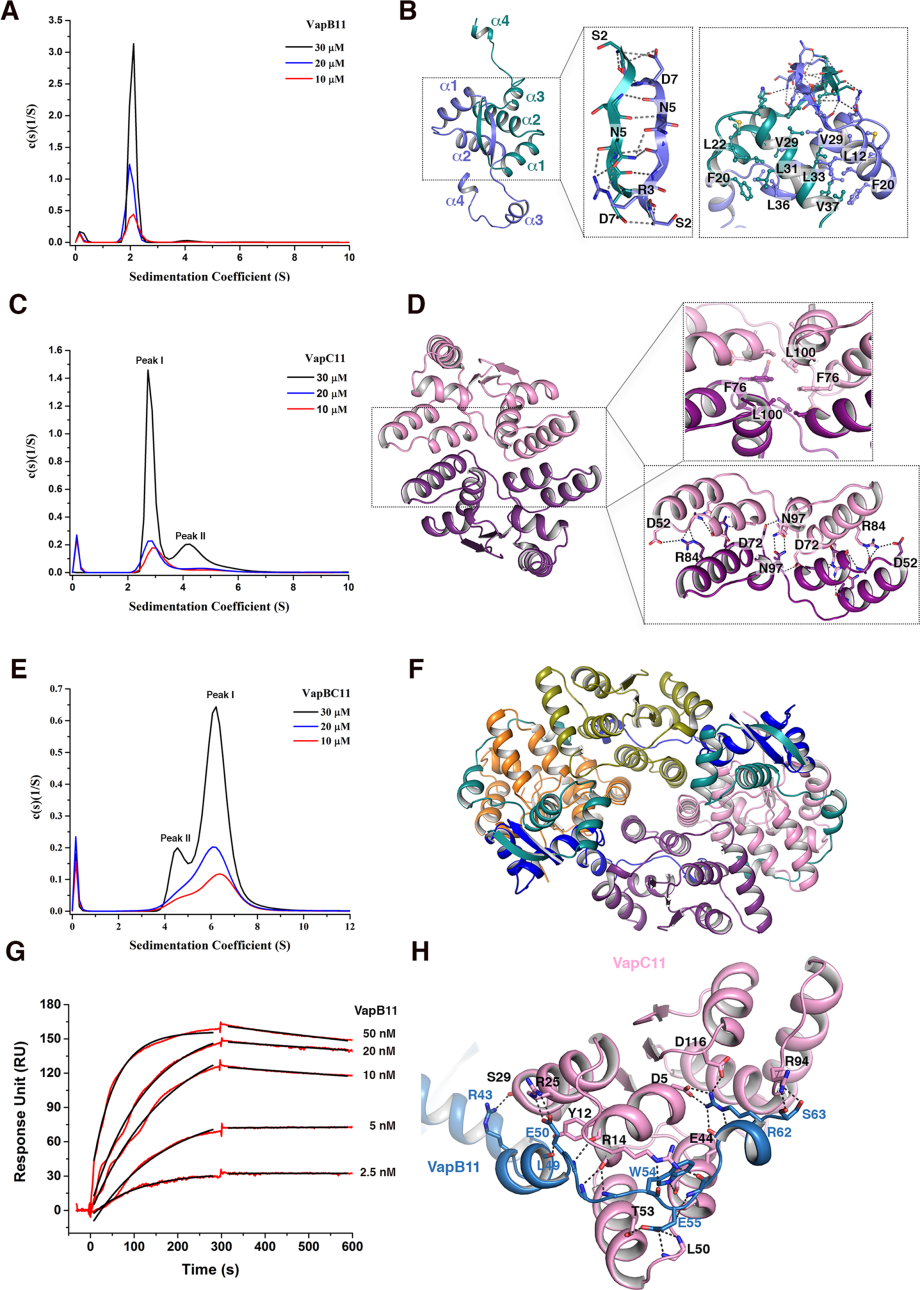
Resolving the oligomeric status of VapB11, VapC11 and VapBC11 TA complex. (**A**) Sedimentation coefficient distribution of VapB11 calculated from sedimentation velocity experiment, showing a major single peak corresponding to homodimeric population at three different concentrations. (**B**) Detailed structural view of VapB11 showing homodimerization. Two chains are shown in purple and green colours. The rectangular box shows cartoon stick presentation for polar hydrogen bond and salt bridge interactions. The square boxes indicate residues involved in non-bonded hydrophobic interactions. (**C**) Sedimentation coefficient distribution for VapC11 showing that VapC11 exists in two oligomeric states corresponding to either homodimer (Peak I) or homotetramer (Peak II). (**D**) Structural organization of VapC11 homodimer. Two chains of a homodimer are shown in pink and purple colours. Square and rectangular boxes indicate residues involved in hydrophobic and polar interactions, respectively. (**E**) Sedimentation coefficient distribution for VapBC11 at three different concentrations. The major peak (Peak II) corresponds to heterooctameric (VapB_4_C_4_11) species, while the minor peak (Peak I) belongs to the heterohexameric population. (**F**) A different view of VapBC11 heterooctameric complex, where antitoxin dimers are facing upwards. (**G**) SPR profile showing association and dissociation profiles of VapB11 and VapC11 interactions. (**H**) A simplified view of VapB11 and VapC11 interactions; monomeric chains of VapB11 (light blue) and VapC11 (pink) are shown. Polar contacts at the interaction interface, including hydrogen bonds and salt bridges, are shown in dotted lines.

To investigate the oligomeric states of the VapBC11 TA complex, we next performed AUC experiments at three different concentrations (10, 20 and 30 μM). The AUC experiments suggest that VapB11 predominantly (∼90%) exists as a homodimer, with an observed *S*_w_ (*S*) of 2.06 ± 0.05 and a frictional ratio of 1.35 ± 0.04 (Figure 4A). The crystal structure reveals that VapB11 oligomerizes via its N-terminal region, burying an interface area of ∼1415 Å^2^ (Δ*G* = −18 kcal/mol), as calculated by PDBePISA (52). The network of interactions in the N-terminal domain of VapB11 is depicted in Figure 4B. The AUC experiments further suggest that VapC11 also predominantly exists as a homodimer, with an observed *S*_w_ (*S*) of 2.99 ± 0.14 and a frictional ratio of 1.27 ± 0.07 (Peak I, Figure 4C). Further, we observed that VapC11 forms a concentration-dependent homotetrameric species that corresponds to the molecular weight of ∼60 kDa (Peak II, Figure 4C). Accordingly, the analysis of the crystal structure revealed that VapC11 forms a homodimer, burying an ∼1220 Å^2^ surface area (Δ*G* = −8 kcal/mol). As shown in Figure 4D, several residues at the dimeric interface, such as Asp52, Asp72, Arg84 and Asn97, interact via hydrogen bonding and salt bridges, whereas hydrophobic residues Phe76 and Leu100 are involved in non-bonded interactions. The structural analysis suggests that the VapC11 homotetrameric population is stabilized by intermolecular interactions from residues such as Arg88 and Asp52, corresponding to α4 and α5 in VapC11 (Supplementary Figure S8). To further understand the oligomeric state(s) of the VapBC11 complex, we performed concentration-dependent titration experiments, but this resulted in visible precipitation, as we had previously observed in the case of the VapBC20 complex (30). Therefore, we performed these experiments with VapBC11 complex purified using a co-expression co-purification strategy. In agreement with the VapBC11 crystal structure, we observed that both VapB11 and VapC11 homodimers interact to form a heterooctamer in solution (VapB_4_C_4_) (Figure 4E and F). The AUC experiments confirmed that VapBC11 complex exists predominantly (80%) as a heterooctamer, with an *S*_w_ (*S*) of 6.20 ± 0.10 at a frictional ratio of 1.20 ± 0.01 (Peak I, Figure 4E). Interestingly, a minor population of ∼75 kDa that most likely corresponds to heterohexameric complex (VapB_2_C_4_) was also observed in the AUC experiments (Peak II, Figure 4E). Taken together, in-solution experiments and the crystal structure have provided valuable insights into the molecular assembly of the VapBC11 complex.

### VapB11 and VapC11 interact with nanomolar range affinity via a multitude of interactions

We next performed SPR to determine the binding affinity and kinetics of VapB11 and VapC11 interactions. Using immobilized VapC11, we observed that VapB11 forms a tight complex with VapC11, with a dissociation constant (*K*_D_) of ∼0.6 nM (Figure 4G). The high association rate (*k*_on_ = 1.15 e^6^ M^−1^ s^−1^) coupled with a slow dissociation rate (*k*_off_ = 1.33 e^−4^ s^−1^) would ensure fast and stable toxin neutralization in the bacteria. The detailed analysis of the crystal structure revealed that each VapBC11 interface buries a large area of ∼1375 Å^2^ (Δ*G* = −7.5 kcal/mol) (Figure 4H). The VapBC11 interface is stabilized by a large network of interactions that includes 24 hydrogen bonds, 18 salt bridges and several non-bonded interactions (Figure 4H). The C-terminal residues of VapB11 (Leu41-Ser63) wrap VapC11, starting from the lateral site towards the active site (Figure 4H). Arg43 and Glu50 residues, corresponding to α3 of VapB11, form salt bridge interactions, respectively, with Ser29 and Arg25 of VapC11 in the lateral site (Figure 4H). We also observed that Leu residues (Leu41, Leu46, Leu47 and Leu49) of VapB11 fit well in the hydrophobic regions of the VapC11 lateral site to form non-bonded interactions. The highly conserved Trp54 residue in VapB11 (Supplementary Figure S9A) is buried in a hydrophobic cavity composed of α1, α3 and α5 helices of VapC11 (Figure 4H). We also observed that Arg14 of VapC11 interacts with the main chain oxygen atoms of Trp54 and Gly6 of VapB11 (Figure 4H). In addition, salt bridge interactions are also formed between Ser63 of VapB11 and Arg94 of VapC11. The highly conserved Arg (i.e. Arg62) in VapB11 forms complex salt bridge interactions with highly conserved active site residues Asp5, Glu44 and Asp116 of VapC11. These interactions consequently overlap with the potential metal ion binding site of VapC11 (Figure 4H). This might explain our observation for a lack of bound metal ion in the VapBC11 crystal structure. Taken together, the binding kinetics and structural data indicate that VapBC11 forms a stable heterooctameric complex in solution.

### VapC11 binds tRNA-LeuT^CAG^ with high affinity

**Figure 5.**
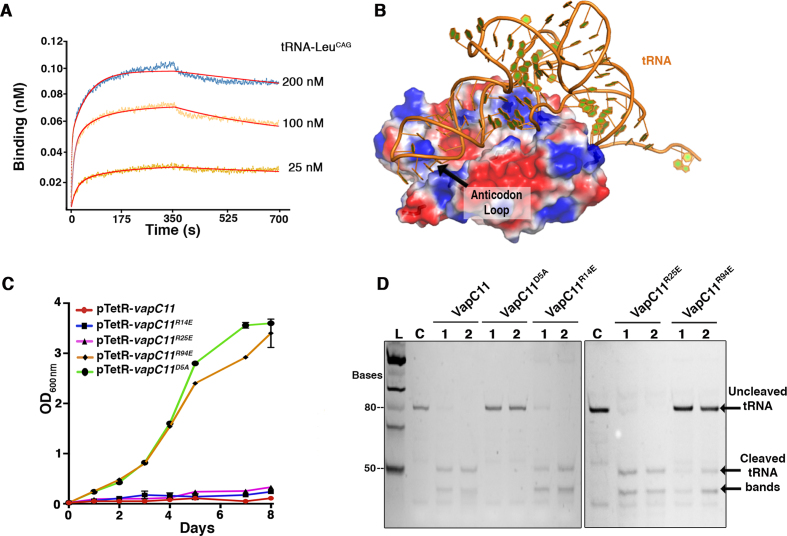
VapC11^D5A^ interacts with tRNA-Leu^CAG^ with a nanomolar range dissociation constant. (**A**) BLI profile of VapC11^D5A^ and tRNA-Leu^CAG^ interactions. The binding sensorgrams, as a nM shift, are shown in different colours for mentioned tRNA concentrations. (**B**) HDOCK-based model for VapC11 and tRNA interactions. Structural information for tRNA-Leu^CAG^ was not available; therefore, we extracted tRNA coordinates from PDB ID: 4V87. This structure was used as a ligand, and docking was performed as per the default parameters in HDOCK. VapC11 binds tRNA with 2:1 stoichiometry. VapC11 and docked tRNA are shown in electrostatic potential and cartoon representation (orange), respectively. (**C**) The growth of strains harbouring either wild-type or single point mutants was determined by measuring absorbance at 600 nm. The expression of proteins in these cultures was induced with the addition of 50 ng/ml of anhydrotetracycline. (**D**) *In vitro* tRNA-Leu^CAG^ cleavage assays were performed with purified VapC11, VapC11^D5A^, VapC11^R14E^, VapC11^R25E^ and VapC11^R94E^. Lanes 1 and 2 indicate 5 and 20 min reaction incubation times for enzymatic reactions, respectively.

VapC11 reportedly cleaves *Msm* tRNA3^Leu-CAG^, tRNA13^Leu-GAG^ and tRNA10^Gln-CTG^*in vitro*, and *Mtb* tRNA3^Leu-CAG^ orthologue (annotated as tRNA-Leu^CAG^ in Mycobrowser https://mycobrowser.epfl.ch) *in vivo* (24). The molecular basis for substrate recognition in VapC toxins is poorly understood, as there are limited studies describing the essential features in molecular recognition of the target by these ribonucleases (44,56). However, the affinity and binding kinetics of VapC toxins with their cellular substrates has not been studied so far. Therefore, we performed binding kinetic experiments of tRNA-Leu^CAG^ using BLI with immobilized VapC11^D5A^. The interaction analysis revealed that VapC11^D5A^ binds to tRNA-Leu^CAG^ with a dissociation constant (*K*_D_) of ∼0.5 nM (Figure 5A), suggesting a tight binding, with association and dissociation rates of *k*_on_ = 1.28 e^6^ M^−1^ s^−1^ and *k*_off_ = 4.25 e^−4^ s^−1^, respectively (Figure 5A). The observed lower *k*_off_ rate could be attributed to the inability of VapC11^D5A^ to cleave its cellular target. We assume that the wild-type protein might have a higher *k*_off_ rate to facilitate faster release of the cleaved product from the active site.

### Arg94 is crucial for binding substrate

Although structures for several VapC toxins have been determined, how they interact with their substrates is poorly understood. Here, we performed VapC11: tRNA-Leu^CAG^ docking using a hybrid docking strategy with the HDOCK server to understand the mode of tRNA recognition and to identify the residue(s) involved in binding substrate (68). The HDOCK server utilizes an FFT-based global docking programme, HDOCKlite, to globally sample putative binding modes. The programme utilizes an improved shape-based, pairwise scoring function. Docking was performed using default parameters, where rotational sampling was performed at 15°, and a spacing of 1.2 Å was adopted for the FFT-based translational search. Since there were no structural data available for any of the VapC11 target tRNAs, we extracted tRNA-Leu^CAG^ from a ribosome structure for use as a substrate template (PDB ID: 4V87). Out of the 100 output models, the top 10 models with high docking scores (−256.16 to −217.40) were further analysed manually. One of the criterion to select the plausible model was to see the positioning of the target cleavage site in the anticodon loop close to the active site in VapC11. Interestingly, one of the top three models met this criterion. The docking results suggested a plausible model of tRNA and VapC interacting with a stoichiometry of 1:2 (Figure 5B). A similar docked pose has also been reported earlier for modelled VapC4 toxin (56). As shown in Figure 5B, the experimentally confirmed cleavage site in the anticodon loop of the tRNA was positioned near to the active site of a monomer in the dimer. Interestingly, the docked model suggested that tRNA might interact with surface-exposed arginine residues such as Arg14, Arg25 and Arg94. In general, Arg residues are known to mediate potential interactions with the nucleic acid phosphate backbone and bases in protein–nucleic acid complex structures (69). To verify the plausible role of Arg14, Arg25 and Arg94 in substrate-RNA recognition, we mutated these positively charged residues to negatively charged Glu to aid electrostatic repulsion. These point variants were overexpressed in *M. bovis* BCG using an anhydrotetracycline-inducible vector, as reported previously (10). We observed that overexpression of VapC11^R94E^ did not result in growth inhibition of *M. bovis* BCG (Figure 5C). However, the growth pattern of *M. bovis* BCG overexpressing VapC11^R14E^ and VapC11^R25E^ was similar to those observed for strains overexpressing wild-type protein (Figure 5C). This Arg94 is positioned in the loop connecting α5-α6 in VapC11 and is highly conserved in other VapC homologues (Supplementary Figure S5). To further verify the *in vitro* growth inhibition results, circular dichroism experiments were performed to see what structural variations, if any, were present in VapC11^R94E^. We observed that the secondary structure contents of VapC11^R94E^ mutant protein was comparable to that observed for VapC11^D5A^, suggesting that the mutant protein adopted a native-like conformation (Supplementary Figure S9B). Next, we compared the tRNA cleavage activity of wild-type protein with various mutant proteins. As shown in Figure 5D, there was a significant reduction in the ribonuclease activity in the case of VapC11^R94E^, hence explaining the observed lack of growth defects associated with its overexpression in *M. bovis* BCG. In accordance with the growth inhibition results, the tRNA cleavage activity of VapC11^R14E^ and VapC11^R25E^ was comparable to that observed for wild-type protein (Figure 5D). These results suggest that Arg94 plays an important role in either tRNA-Leu^CAG^ binding or cleavage. Since Arg94 is situated ∼6 Å distant from the active site (Asp98 OD2 to Arg94 Cα), the observed reduction in activity is most likely due to the reduced affinity for the tRNA substrate. As observed in the above BLI experiments, VapC11^D5A^ interacts with tRNA with a nanomolar dissociation constant; therefore, to assess the contribution of Arg94 we created the VapC11^R94E^ mutation in the background of the VapC11^D5A^ construct. The BLI experiments suggest that VapC11^R94E, D5A^ lost its affinity for tRNA substrate, thus validating that Arg94 is crucial for binding tRNA substrate (Supplementary Figure S9C).

### VapB11-derived peptides inhibit VapC11-dependent cleavage of tRNA-Leu^CAG^

**Figure 6.**
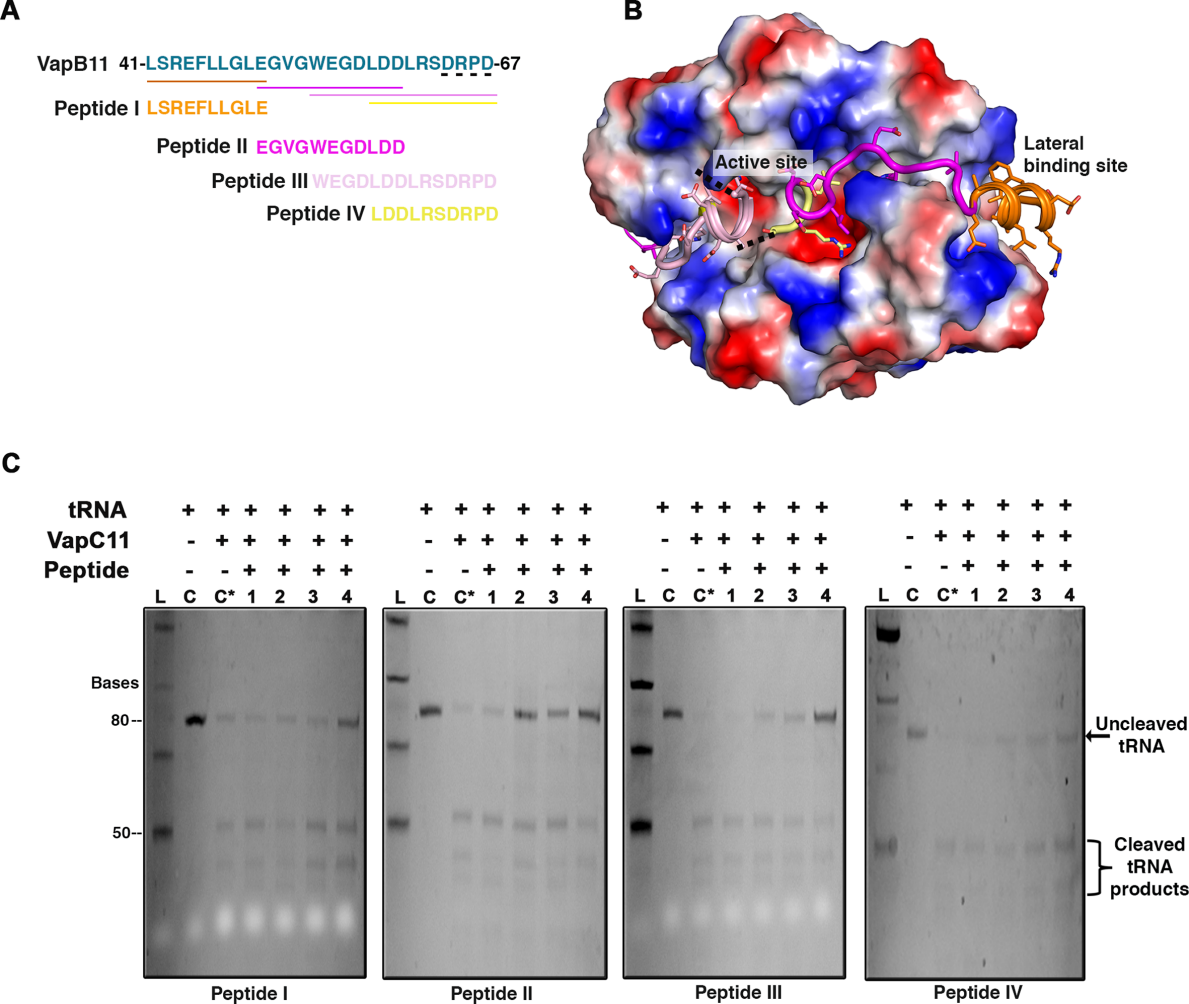
Antitoxin-derived peptide inhibitors block tRNA cleavage. (**A**) C-terminal VapB11 sequence involved in interactions with VapC11 (Leu41 to Asp67) is shown in green shade (top). Four overlapping designed peptide sequences aligned to the VapB11 C-terminal toxin binding region are highlighted. (**B**) Structural representation of VapC11 (electrostatic representation)- and VapB11 (cartoon representation)-derived peptide regions are shown in the same colour as in panel (A). The four residues in the C-terminal of VapB11 that could not be resolved in the crystal structure are shown in black dotted line. (**C**) Panel showing *in vitro* ribonuclease assay in the presence of four different designed peptides. 1, 2, 3 and 4 represent the final concentrations of peptide inhibitors as 10, 100, 250 and 1000 μM, respectively. Abbreviations: C, negative control for tRNA-Leu^CAG^ cleavage; C*, positive control for tRNA-Leu^CAG^ cleavage; L, Low range ssRNA ladder.

Since toxins are neutralized by their cognate antitoxins, one of the attractive strategies for killing microbes is to activate endogenous toxins by disrupting their interaction with cognate antitoxins (70). Our findings demonstrated that replacement of the VapBC11 TA locus with the hygromycin resistance gene significantly attenuated the growth of *Mtb in vivo*. This suggests the presence of a signalling mechanism that results in the activation of VapBC11 and the presence of free toxin result in the metabolic slowdown that enables *Mtb* to adapt to *in vivo* stress conditions. Considering these findings, we employed a novel approach to design structure-guided peptide-based inhibitors in an attempt to block VapC11 ribonuclease activity. Four overlapping peptides of 10–14 residues (Figure 6A and B) were designed, and VapC11 ribonuclease activity was measured *in vitro* in the presence of these peptides using tRNA-Leu^CAG^ substrate. Interestingly, all four peptides were able to inhibit VapC11 ribonuclease activity in a dose-dependent manner (Figure 6C). There was a reduction in the cleaved products upon addition of the peptide inhibitors. These results suggest that all four peptides may sterically occlude binding of tRNA to the VapC11 active site. Interestingly, peptide-I, which was designed to bind at the lateral site located away from the active site, was also able to inhibit VapC11 ribonuclease activity. This observation also supports the above-discussed docking-based structural model where tRNA binds at the lateral site (Figure 5B). Taken together, we demonstrate that peptide-based inhibitors are capable of inhibiting the ribonuclease activity associated with VapC11.

## DISCUSSION

The abundance of TA systems in the *Mtb* genome warrants studies to decipher their role in bacterial physiology (11,71). In the present study, we functionally and structurally characterized the VapBC11 TA system from *Mtb*, as its overexpression inhibits growth of mycobacterial species and its expression is upregulated in response to several stress conditions, including exposure to various antibiotics (10,25). RNA-Seq analysis revealed that the expression profiles of overexpression strains were highly similar to bacteria exposed to different stress conditions, such as nutritional stress, low oxygen and drugs. While expression of VapC11 may directly affect the synthesis of several proteins, the large-scale changes in the expression profile can partly be explained by a concerted effect from overexpression of extracytoplasmic function sigma factor *sigE*, transcriptional regulators (*merR, asR, whiB6*), cellular proteases (*clpC2, mycP4*), chromosomal toxins, several annotated transcription regulatory proteins and a large set of hypothetical proteins. In accordance with the previous results, overexpression of VapC11 also induces bacteriostasis, and the VapBC11 TA system is dispensable for adaptation of *Mtb* to *in vitro* stress conditions (10). However, the mutant was severely attenuated for growth *in vivo*, and this growth defect was more prominent in the chronic stage of infection. It appears likely that a reduction in cellular metabolism is crucial for *Mtb* survival in the host. In a recent report, high-affinity mutants of CarD were shown to enhance the *Mtb* growth rate *in vitro* but had poor growth in a mouse model, suggesting that *Mtb* growth is optimized for *in vivo* survival (72). This report and the data presented here imply that a reduction in bacterial metabolism enables the bacterium to persist and adapt to different stress conditions.

**Figure 7.**
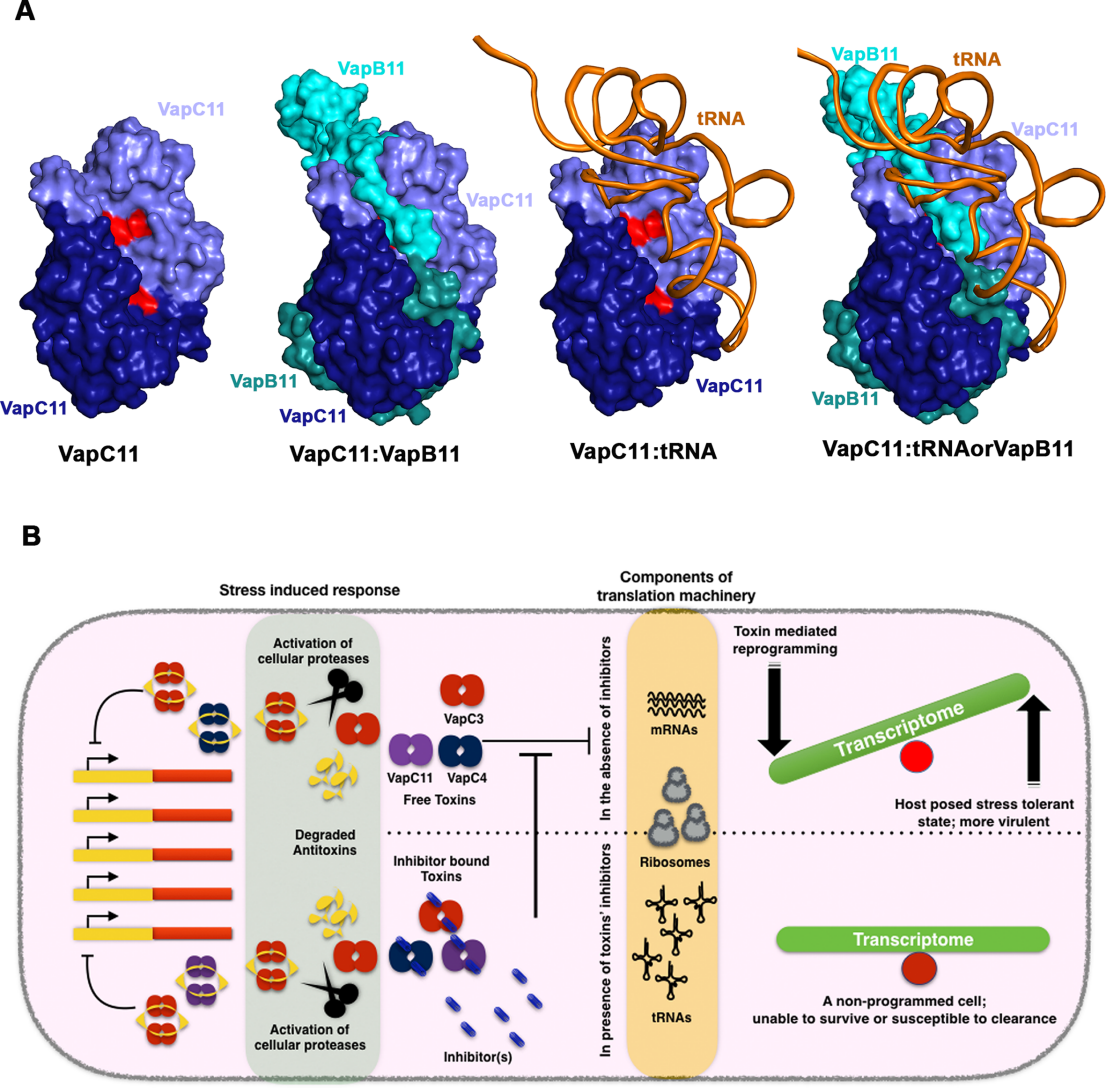
A plausible model for VapC11–tRNA (toxin–target) interactions and a potential strategy for designing toxin inhibitory peptides/small molecules. (**A**) Structural models showing VapC11, VapBC11 complex, modelled VapC11–tRNA complex and a superimposed structure showing overlap in antitoxin and tRNA-binding sites on VapC11. The active sites of VapC11 homodimer are coloured in red. Different chains are shown in different colours with their respective labels. (**B**) An overall model showing effect(s) of toxin expression and a potential strategy to design inhibitors targeted against VapC toxins. In stress-induced responses, the antitoxin molecules are degraded by cellular proteases. The resulting free toxins inhibit translation by degrading either mRNA or rRNA or tRNA, thereby reprogramming cells to reduce cell growth to facilitate cell survival in the presence of antibiotic- or host-induced stress. A novel approach is to design inhibitors targeting ribonuclease activity that might render bacterial cells susceptible to killing.

The crystal structure analysis of VapBC11 revealed that it forms a closed heterooctameric complex. The two distinct DNA-binding RHH motifs in the VapBC11 crystal structure are separated by a spatial distance of ∼60 Å, comparable to that observed in the FitAB–DNA complex (64). Though antitoxins are reported to interact directly with *cis*-elements, the formation of the TA complex bridges two DNA-binding domains, facilitating the optimal positioning to recognize two inverted repeat sequences in the operator region. We speculate that this arrangement might improve the binding affinity of the TA-operator DNA complex (66,73). From a substrate recognition point of view, the existing structural data indicate that residues that are present on the surface closer to the active site might play a crucial role in substrate binding. Docking studies suggest that tRNA substrate interacts with VapC11 in a 1:2 stoichiometry and competes with VapB11 for overlapping binding sites (Figure 7A). Our data suggest that VapC11^D5A^ toxin interacts with tRNA-Leu^CAG^ substrate or VapB11 antitoxin with a comparable dissociation constant. Upon functional and biochemical characterization, we conclude that Arg94 is crucial for ribonuclease activity and probably aids in substrate binding. Similarly, using random site-directed mutagenesis, Arg93 in *Haemophilus influenza* VapC1 has been shown to be important for the ribonuclease activity (74). The essentiality of VapC toxins for survival in hosts inspired us to design structure-guided peptide-based inhibitors to block ribonuclease activity associated with VapC11. We propose that inhibitors that block VapC toxins may prevent metabolic changes that enable *Mtb* to survive in the host, and hence, can be exploited as drug targets (Figure 7B). Interestingly, all four peptides spanning a C-terminal toxin-binding antitoxin domain inhibited the tRNA substrate cleavage in a concentration-dependent manner. Of special interest was peptide-I, which was designed to bind at the lateral site on VapC11 and inhibited tRNA cleavage. Previously, this lateral site has been exploited to design inhibitors targeted to disrupt toxin–antitoxin interactions, thereby activating endogenous toxin, resulting in growth arrest (32,33). In these studies, peptide inhibitors were successful in preventing TA interactions, and the cleavage assays were performed using synthetic fluorescent RNA substrates. However, our data suggest that even peptides targeted to bind at the TA interface may inhibit cleavage of the natural substrate. Therefore, the data presented in this study highlight the importance of using native tRNA substrates in these inhibitor assays to identify small molecule inhibitors. Based on these observations, we propose a plausible model for tRNA binding, where VapB11, in addition to occluding the toxin active site, also overlaps with the tRNA-binding region on VapC11. The proposed strategy for developing potential inhibitors against *Mtb* by blocking toxin activity is counterintuitive and a novel proposition.

To conclude, the results presented in this study have clarified that free VapC11 in bacteria results in major changes in transcriptional profiles that enable the bacterium to survive in the host. We have identified key residues that are important for VapBC11 TA interactions. Furthermore, we propose a plausible model for substrate recognition by the VapC11 toxin. Future studies should include determining the structure of VapC11 in complex with its tRNA substrate. Since VapC toxins (VapBC3, VapBC4 and VapBC11) are indispensable for growth *in vivo* and share common structural features, inhibitor(s) blocking multiple ribonucleases can be designed. We speculate that these inhibitors would also be active against drug-resistant TB and be effective in tackling the problem of drug-resistance.

## DATA AVAILABILITY

The crystal structure coordinates and structure factor file have been deposited in RCSB PDB under the PDB ID: 6A7V.

RNA-Seq data have been deposited in the NCBI Gene Expression Omnibus (GEO) database with accession code GSE116861.

## Supplementary Material

Supplementary Data
