# Supplementary material for: Structural, functional and biological insights into the role of Mycobacterium tuberculosis VapBC11 toxin–antitoxin system: targeting a tRNase to tackle mycobacterial adaptation
Source: Nucleic Acids Res. 2018 Oct 17;46(21):11639–55. doi: 10.1093/nar/gky924 (PMC6265470; doi:10.1093/nar/gky924)
Supplement: Supplementary Data [file gky924_supplemental_files.zip › Table S2_VapBC11.docx]

**Supplementary Table S2**

| **Gene Name** | **Gene ID** | **log2FoldChange** | **P value** |
| --- | --- | --- | --- |
| **Cell wall and cell processes** |  |  |  |
| ansP2 | Rv0346c | -2.30447 | 0.00005 |
| arsC | Rv2643 | 2.98479 | 0.00005 |
| ctpC | Rv3270 | 3.66621 | 0.00005 |
| ctpE | Rv0908 | -2.34175 | 0.0008 |
| cut1 | Rv1758 | 2.22535 | 0.03575 |
| cut3 | Rv3451 | 3.02794 | 0.00005 |
| cut5b | Rv3724B | 2.11985 | 0.0112 |
| cycA | Rv1704c | -2.10654 | 0.00005 |
| drrA | Rv2936 | -2.14035 | 0.0003 |
| embA | Rv3794 | -2.35612 | 0.0004 |
| esxG | Rv0287 | -5.03045 | 0.00005 |
| esxH | Rv0288 | -3.86737 | 0.00005 |
| esxJ | Rv1038c | 2.06351 | 0.0054 |
| esxK | Rv1197 | -2.42892 | 0.00005 |
| esxL | Rv1198 | -2.7393 | 0.00005 |
| fic | Rv3641c | 3.13009 | 0.00005 |
| ftsW | Rv2154c | -2.31322 | 0.00165 |
| ftsZ | Rv2150c | -2.86821 | 0.00005 |
| glnQ | Rv2564 | -2.48636 | 0.00005 |
| lepB | Rv2903c | 2.51457 | 0.00005 |
| lgt | Rv1614 | -3.15942 | 0.00005 |
| lppK | Rv2116 | 2.90895 | 0.00005 |
| lppU | Rv2784c | 3.2576 | 0.00005 |
| lppX | Rv2945c | -3.99429 | 0.00005 |
| lpqB | Rv3244c | -2.57076 | 0.00745 |
| lpqL | Rv0418 | -2.23579 | 0.00005 |
| lpqS | Rv0847 | 3.50003 | 0.00005 |
| lprC | Rv1275 | -3.05828 | 0.02905 |
| lprJ | Rv1690 | 2.86918 | 0.00005 |
| lprP | Rv0962c | 2.31414 | 0.0023 |
| ltp1 | Rv2790c | -3.03648 | 0.00005 |
| manB | Rv3264c | -3.05192 | 0.00005 |
| mgtE | Rv0362 | 2.16029 | 0.00005 |
| mmpL10 | Rv1183 | -2.58323 | 0.00005 |
| mpt53 | Rv2878c | 2.80799 | 0.00005 |
| mpt70 | Rv2875 | 3.87118 | 0.00005 |
| mptA | Rv2174 | -2.20144 | 0.0045 |
| murG | Rv2153c | -2.23194 | 0.01445 |
| narK1 | Rv2329c | -2.93587 | 0.00005 |
| narK3 | Rv0261c | 2.97378 | 0.0003 |
| phoY1 | Rv3301c | -2.3972 | 0.00005 |
| ponA1 | Rv0050 | -2.64932 | 0.00005 |
| pstA2 | Rv0936 | -2.82999 | 0.0003 |
| pstC1 | Rv0935 | -2.01278 | 0.00005 |
| pstS1 | Rv0934 | -3.0043 | 0.00005 |
| rpfC | Rv1884c | 2.21016 | 0.00005 |
| rpfE | Rv2450c | 3.4604 | 0.00005 |
| Rv0051 | Rv0051 | -2.84764 | 0.0036 |
| Rv0073 | Rv0073 | -3.32608 | 0.00005 |
| Rv0229c | Rv0229c | 2.01799 | 0.0163 |
| Rv0435c | Rv0435c | -2.1361 | 0.00105 |
| Rv0463 | Rv0463 | -2.50321 | 0.00015 |
| Rv0519c | Rv0519c | -2.33126 | 0.00005 |
| Rv0528 | Rv0528 | -2.7076 | 0.00035 |
| Rv0531 | Rv0531 | -2.41805 | 0.00005 |
| Rv0584 | Rv0584 | 2.22495 | 0.00005 |
| Rv0621 | Rv0621 | 3.1485 | 0.00005 |
| Rv0622 | Rv0622 | 2.54933 | 0.00005 |
| Rv0841 | Rv0841 | 4.64023 | 0.0186 |
| Rv0901 | Rv0901 | -2.35018 | 0.00005 |
| Rv1081c | Rv1081c | 2.07636 | 0.0002 |
| Rv1087A | Rv1087A | 3.684 | 0.0004 |
| Rv1132 | Rv1132 | -2.37297 | 0.00005 |
| Rv1184c | Rv1184c | -2.10294 | 0.00005 |
| Rv1410c | Rv1410c | -2.09896 | 0.00085 |
| Rv1457c | Rv1457c | -2.01083 | 0.0266 |
| Rv1481 | Rv1481 | -2.69385 | 0.00005 |
| Rv1735c | Rv1735c | 4.47015 | 0.0017 |
| Rv1986 | Rv1986 | 2.26556 | 0.00005 |
| Rv2025c | Rv2025c | 2.79153 | 0.00005 |
| Rv2077c | Rv2077c | 3.4592 | 0.00005 |
| Rv2197c | Rv2197c | -2.07072 | 0.0155 |
| Rv2199c | Rv2199c | -2.37242 | 0.00005 |
| Rv2203 | Rv2203 | 2.66472 | 0.00005 |
| Rv2253 | Rv2253 | 4.35432 | 0.00005 |
| Rv2254c | Rv2254c | 4.04143 | 0.00005 |
| Rv2609c | Rv2609c | -3.88499 | 0.00015 |
| Rv2719c | Rv2719c | 3.58186 | 0.00005 |
| Rv2723 | Rv2723 | 2.33384 | 0.00005 |
| Rv2732c | Rv2732c | -2.53965 | 0.018 |
| Rv2864c | Rv2864c | -2.05523 | 0.00005 |
| Rv2876 | Rv2876 | 2.06 | 0.00005 |
| Rv2877c | Rv2877c | 2.71901 | 0.00005 |
| Rv3104c | Rv3104c | -2.94882 | 0.0018 |
| Rv3193c | Rv3193c | -2.2916 | 0.00005 |
| Rv3289c | Rv3289c | 2.56385 | 0.0001 |
| Rv3312A | Rv3312A | -3.0105 | 0.0003 |
| Rv3453 | Rv3453 | 2.41503 | 0.0011 |
| Rv3693 | Rv3693 | -2.03368 | 0.00005 |
| Rv3789 | Rv3789 | 2.27976 | 0.0001 |
| Rv3807c | Rv3807c | -3.65916 | 0.00005 |
| Rv3848 | Rv3848 | 4.14165 | 0.00005 |
| Rv3857c | Rv3857c | 2.05055 | 0.00075 |
| secE1 | Rv0638 | 3.59749 | 0.00005 |
| smc | Rv2922c | -2.02423 | 0.00005 |
| TB8.4 | Rv1174c | 2.613 | 0.00005 |
| ldtA | Rv0116c | 2.11756 | 0.00005 |
| eccD3 | Rv0290 | -4.24394 | 0.00005 |
| eccE3 | Rv0292 | -3.97705 | 0.0041 |
| eccC5 | Rv1783 | -3.54871 | 0.00005 |
| fxsA | Rv2053c | -2.29748 | 0.00005 |
| mpa | Rv2115c | -2.33123 | 0.00005 |
| ubiA | Rv3806c | -3.59598 | 0.00005 |
| eccCa1 | Rv3870 | -2.70727 | 0.0003 |
| eccD1 | Rv3877 | -2.60782 | 0.0232 |
| eccE2 | Rv3885c | -2.40844 | 0.029 |
| **Conserved Hypothetical** |  |  |  |
| lsr2 | Rv3597c | 2.35775 | 0.00005 |
| Rv0047c | Rv0047c | 3.82052 | 0.00005 |
| Rv0060 | Rv0060 | 2.54807 | 0.00005 |
| Rv0074 | Rv0074 | -2.58058 | 0.00005 |
| Rv0122 | Rv0122 | 2.65971 | 0.0312 |
| Rv0193c | Rv0193c | 2.13289 | 0.00005 |
| Rv0250c | Rv0250c | -2.05652 | 0.00015 |
| Rv0259c | Rv0259c | 2.1336 | 0.0067 |
| Rv0263c | Rv0263c | 2.13441 | 0.00005 |
| Rv0264c | Rv0264c | 3.66137 | 0.00005 |
| Rv0268c | Rv0268c | 3.10214 | 0.00005 |
| Rv0340 | Rv0340 | 2.72576 | 0.00005 |
| Rv0366c | Rv0366c | 5.13392 | 0.00005 |
| Rv0367c | Rv0367c | 4.28328 | 0.00005 |
| Rv0368c | Rv0368c | 2.18369 | 0.00035 |
| Rv0378 | Rv0378 | 2.54639 | 0.02025 |
| Rv0421c | Rv0421c | -2.28408 | 0.00355 |
| Rv0448c | Rv0448c | 2.8395 | 0.001 |
| Rv0516c | Rv0516c | 2.59588 | 0.00005 |
| Rv0607 | Rv0607 | 2.54717 | 0.00025 |
| Rv0610c | Rv0610c | 2.17561 | 0.00005 |
| Rv0613c | Rv0613c | -3.02378 | 0.0002 |
| Rv0634A | Rv0634A | -2.59865 | 0.00005 |
| Rv0739 | Rv0739 | 3.21018 | 0.00005 |
| Rv0749A | Rv0749A | 3.80346 | 0.0076 |
| Rv0750 | Rv0750 | 3.74089 | 0.00005 |
| Rv0793 | Rv0793 | 2.55675 | 0.01525 |
| Rv0799c | Rv0799c | -2.51519 | 0.0016 |
| Rv0801 | Rv0801 | -2.88852 | 0.0263 |
| Rv0826 | Rv0826 | 2.8357 | 0.00005 |
| Rv0918 | Rv0918 | 3.35714 | 0.0009 |
| Rv0959 | Rv0959 | -2.3038 | 0.002 |
| Rv0963c | Rv0963c | 2.58816 | 0.00005 |
| Rv0968 | Rv0968 | 2.45789 | 0.00005 |
| Rv0990c | Rv0990c | 2.91308 | 0.00005 |
| Rv1000c | Rv1000c | 2.47228 | 0.00005 |
| Rv1025 | Rv1025 | -3.15799 | 0.0012 |
| Rv1044 | Rv1044 | 4.8797 | 0.00005 |
| Rv1045 | Rv1045 | 2.4471 | 0.00095 |
| Rv1048c | Rv1048c | 2.72507 | 0.00005 |
| Rv1052 | Rv1052 | 2.28285 | 0.00435 |
| Rv1057 | Rv1057 | 4.94004 | 0.00005 |
| Rv1069c | Rv1069c | -2.72024 | 0.00055 |
| Rv1265 | Rv1265 | 4.35251 | 0.00005 |
| Rv1277 | Rv1277 | 2.89021 | 0.00075 |
| Rv1372 | Rv1372 | 2.44209 | 0.00065 |
| Rv1374c | Rv1374c | 3.03461 | 0.00005 |
| Rv1378c | Rv1378c | 4.27371 | 0.00005 |
| Rv1439c | Rv1439c | 3.5658 | 0.00005 |
| Rv1498A | Rv1498A | 2.05967 | 0.00015 |
| Rv1503c | Rv1503c | 3.61942 | 0.00005 |
| Rv1545 | Rv1545 | -2.38828 | 0.0015 |
| Rv1546 | Rv1546 | -2.13526 | 0.00005 |
| Rv1590 | Rv1590 | -3.76622 | 0.004 |
| Rv1638A | Rv1638A | -3.24799 | 0.0001 |
| Rv1645c | Rv1645c | 5.00428 | 0.00005 |
| Rv1680 | Rv1680 | -2.23042 | 0.01045 |
| Rv1691 | Rv1691 | 2.27897 | 0.0025 |
| Rv1697 | Rv1697 | -2.75075 | 0.00005 |
| Rv1734c | Rv1734c | 4.83128 | 0.00005 |
| Rv1752 | Rv1752 | -3.46147 | 0.00005 |
| Rv1772 | Rv1772 | 2.39163 | 0.00005 |
| Rv1804c | Rv1804c | 3.04588 | 0.0011 |
| Rv1805c | Rv1805c | 2.69052 | 0.00005 |
| Rv1813c | Rv1813c | 4.05106 | 0.00005 |
| Rv1831 | Rv1831 | 2.7194 | 0.00005 |
| Rv1847 | Rv1847 | 2.23591 | 0.00005 |
| Rv1871c | Rv1871c | -2.15696 | 0.00005 |
| Rv1875 | Rv1875 | 2.01646 | 0.00005 |
| Rv1888A | Rv1888A | 2.68176 | 0.01895 |
| Rv1894c | Rv1894c | 2.49743 | 0.00005 |
| Rv1929c | Rv1929c | 3.61891 | 0.00005 |
| Rv1948c | Rv1948c | 2.53143 | 0.0009 |
| Rv1950c | Rv1950c | 2.40842 | 0.03405 |
| Rv1954c | Rv1954c | 5.66721 | 0.00005 |
| Rv1957 | Rv1957 | 3.76048 | 0.00025 |
| Rv1958c | Rv1958c | 2.60847 | 0.00315 |
| Rv1961 | Rv1961 | 3.36457 | 0.00005 |
| Rv1989c | Rv1989c | 6.11046 | 0.00005 |
| Rv1995 | Rv1995 | 2.04647 | 0.0017 |
| Rv2011c | Rv2011c | 5.43682 | 0.00005 |
| Rv2012 | Rv2012 | 3.51286 | 0.00005 |
| Rv2019 | Rv2019 | 3.39328 | 0.0151 |
| Rv2020c | Rv2020c | 3.2167 | 0.00005 |
| Rv2022c | Rv2022c | 3.70999 | 0.00005 |
| Rv2033c | Rv2033c | -2.30982 | 0.00005 |
| Rv2035 | Rv2035 | 4.71907 | 0.00005 |
| Rv2114 | Rv2114 | 2.09144 | 0.00005 |
| Rv2132 | Rv2132 | 2.43047 | 0.00005 |
| Rv2147c | Rv2147c | -2.29514 | 0.00005 |
| Rv2166c | Rv2166c | 2.05298 | 0.00005 |
| Rv2228c | Rv2228c | -2.4005 | 0.0304 |
| Rv2255c | Rv2255c | 2.48426 | 0.00005 |
| Rv2256c | Rv2256c | 2.25748 | 0.00005 |
| Rv2269c | Rv2269c | 4.42518 | 0.00175 |
| Rv2293c | Rv2293c | 2.96469 | 0.00005 |
| Rv2307B | Rv2307B | 2.39542 | 0.002 |
| Rv2307D | Rv2307D | 3.97906 | 0.00145 |
| Rv2309A | Rv2309A | 2.07926 | 0.01835 |
| Rv2312 | Rv2312 | 2.82469 | 0.00005 |
| Rv2314c | Rv2314c | -2.40023 | 0.0003 |
| Rv2315c | Rv2315c | -2.33466 | 0.00065 |
| Rv2325c | Rv2325c | -2.36581 | 0.0154 |
| Rv2413c | Rv2413c | 2.81124 | 0.00005 |
| Rv2415c | Rv2415c | 2.45952 | 0.0001 |
| Rv2425c | Rv2425c | -2.40935 | 0.0008 |
| Rv2426c | Rv2426c | -2.26652 | 0.00105 |
| Rv2451 | Rv2451 | 2.85624 | 0.00005 |
| Rv2466c | Rv2466c | 3.84508 | 0.00005 |
| Rv2468c | Rv2468c | -2.07297 | 0.00025 |
| Rv2472 | Rv2472 | 2.67024 | 0.00005 |
| Rv2474c | Rv2474c | -2.391 | 0.00005 |
| Rv2491 | Rv2491 | 2.20781 | 0.0467 |
| Rv2515c | Rv2515c | 2.3437 | 0.00005 |
| Rv2516c | Rv2516c | 2.58248 | 0.00005 |
| Rv2554c | Rv2554c | -2.80438 | 0.02765 |
| Rv2567 | Rv2567 | -2.22819 | 0.0131 |
| Rv2578c | Rv2578c | 2.02696 | 0.0004 |
| Rv2660c | Rv2660c | 7.12706 | 0.0007 |
| Rv2661c | Rv2661c | 5.00837 | 0.00005 |
| Rv2663 | Rv2663 | 2.58784 | 0.00045 |
| Rv2664 | Rv2664 | 2.64825 | 0.00075 |
| Rv2665 | Rv2665 | 2.43775 | 0.00005 |
| Rv2707 | Rv2707 | 2.68594 | 0.00005 |
| Rv2712c | Rv2712c | 2.22902 | 0.00005 |
| Rv2717c | Rv2717c | 2.52704 | 0.00005 |
| Rv2722 | Rv2722 | 2.72348 | 0.00285 |
| Rv2730 | Rv2730 | 2.65503 | 0.02025 |
| Rv2734 | Rv2734 | 2.34102 | 0.00005 |
| Rv2762c | Rv2762c | -2.65962 | 0.0402 |
| Rv2811 | Rv2811 | 2.34601 | 0.03445 |
| Rv2816c | Rv2816c | -2.00542 | 0.00005 |
| Rv2817c | Rv2817c | -2.22563 | 0.00005 |
| Rv2821c | Rv2821c | -2.28479 | 0.01335 |
| Rv2824c | Rv2824c | 2.07773 | 0.01725 |
| Rv2840c | Rv2840c | 2.29096 | 0.00005 |
| Rv2844 | Rv2844 | -2.48516 | 0.0018 |
| Rv2908c | Rv2908c | 2.35042 | 0.00005 |
| Rv2990c | Rv2990c | 3.12759 | 0.00005 |
| Rv2991 | Rv2991 | 2.27964 | 0.00005 |
| Rv3030 | Rv3030 | -2.15062 | 0.00635 |
| Rv3031 | Rv3031 | -2.51723 | 0.00005 |
| Rv3040c | Rv3040c | -2.23399 | 0.01705 |
| Rv3054c | Rv3054c | 4.34337 | 0.00005 |
| Rv3067 | Rv3067 | 2.70661 | 0.00005 |
| Rv3074 | Rv3074 | 4.57123 | 0.00005 |
| Rv3094c | Rv3094c | -2.49773 | 0.00015 |
| Rv3098c | Rv3098c | 2.41499 | 0.001 |
| Rv3108 | Rv3108 | 3.58488 | 0.00005 |
| Rv3126c | Rv3126c | 2.91162 | 0.0004 |
| Rv3180c | Rv3180c | 2.82262 | 0.00005 |
| Rv3181c | Rv3181c | 2.41757 | 0.00015 |
| Rv3182 | Rv3182 | 6.74784 | 0.00005 |
| Rv3188 | Rv3188 | 4.62836 | 0.00005 |
| Rv3189 | Rv3189 | 4.82964 | 0.00015 |
| Rv3195 | Rv3195 | 2.82743 | 0.00005 |
| Rv3284 | Rv3284 | -2.63671 | 0.0172 |
| Rv3351c | Rv3351c | 2.79581 | 0.00035 |
| Rv3354 | Rv3354 | 2.01483 | 0.00005 |
| Rv3394c | Rv3394c | 3.26779 | 0.0072 |
| Rv3395c | Rv3395c | 5.13155 | 0.00005 |
| Rv3424c | Rv3424c | 3.47428 | 0.00005 |
| Rv3463 | Rv3463 | 2.91371 | 0.00005 |
| Rv3488 | Rv3488 | 3.26597 | 0.00005 |
| Rv3603c | Rv3603c | 2.30912 | 0.00085 |
| Rv3613c | Rv3613c | -2.29912 | 0.0346 |
| Rv3639c | Rv3639c | 2.25234 | 0.00235 |
| Rv3642c | Rv3642c | 3.37839 | 0.00005 |
| Rv3643 | Rv3643 | 4.82454 | 0.04445 |
| Rv3659c | Rv3659c | 2.34672 | 0.0144 |
| Rv3662c | Rv3662c | -2.13786 | 0.0051 |
| Rv3678c | Rv3678c | -2.30465 | 0.00005 |
| Rv3714c | Rv3714c | 2.81418 | 0.00005 |
| Rv3740c | Rv3740c | 2.11893 | 0.00565 |
| Rv3745c | Rv3745c | 2.70787 | 0.0156 |
| Rv3749c | Rv3749c | 2.27005 | 0.00005 |
| Rv3771c | Rv3771c | 2.38349 | 0.00005 |
| Rv3776 | Rv3776 | 3.50452 | 0.00005 |
| Rv3813c | Rv3813c | -2.7832 | 0.00005 |
| Rv3860 | Rv3860 | 4.14751 | 0.00015 |
| Rv3861 | Rv3861 | 3.55935 | 0.00675 |
| Rv3896c | Rv3896c | 2.41626 | 0.00005 |
| TB27.3 | Rv0577 | 2.20913 | 0.00005 |
| espG3 | Rv0289 | -4.13743 | 0.00005 |
| hadA | Rv0635 | 2.8938 | 0.00125 |
| csoR | Rv0967 | 2.96022 | 0.00005 |
| prpD | Rv1130 | 5.03284 | 0.00005 |
| rseA | Rv1222 | 2.07203 | 0.00005 |
| mctB | Rv1698 | -2.26852 | 0.00005 |
| mazF6 | Rv1991c | 5.25029 | 0.00005 |
| pafC | Rv2095c | -2.69604 | 0.0296 |
| pafB | Rv2096c | -2.09062 | 0.00005 |
| dop | Rv2112c | -2.0884 | 0.00005 |
| espG1 | Rv3866 | -2.24626 | 0.00005 |
| eccA1 | Rv3868 | -2.01954 | 0.00105 |
| eccCb1 | Rv3871 | -2.00308 | 0.00005 |
| eccA2 | Rv3884c | -2.09174 | 0.00005 |
| **Information pathways** |  |  |  |
| alaS | Rv2555c | -2.39794 | 0.00005 |
| alkA | Rv1317c | 4.01952 | 0.00005 |
| dnaE2 | Rv3370c | 3.18081 | 0.00005 |
| efp | Rv2534c | 2.44327 | 0.00005 |
| end | Rv0670 | -2.03093 | 0.00005 |
| gatA | Rv3011c | -2.17581 | 0.00005 |
| greA | Rv1080c | 3.58176 | 0.00005 |
| gyrA | Rv0006 | -2.70359 | 0.00005 |
| helZ | Rv2101 | -2.63895 | 0.00005 |
| ileS | Rv1536 | 2.47193 | 0.00005 |
| infC | Rv1641 | 2.48121 | 0.00005 |
| nrdF1 | Rv1981c | -2.52708 | 0.00005 |
| nrdH | Rv3053c | 2.46045 | 0.00005 |
| nrdI | Rv3052c | 2.27568 | 0.00005 |
| nusG | Rv0639 | 2.47086 | 0.00005 |
| ogt | Rv1316c | 4.49378 | 0.00345 |
| pheT | Rv1650 | -2.13381 | 0.00005 |
| prfA | Rv1299 | 2.04228 | 0.00035 |
| radA | Rv3585 | 2.35814 | 0.00005 |
| recN | Rv1696 | -2.08812 | 0.00005 |
| rimJ | Rv0995 | -2.14792 | 0.00005 |
| rimM | Rv2907c | 2.67563 | 0.00005 |
| rnpA | Rv3923c | 2.85232 | 0.00875 |
| rplC | Rv0701 | 2.66999 | 0.001 |
| rplJ | Rv0651 | 3.12502 | 0.00005 |
| rplK | Rv0640 | 2.36803 | 0.00005 |
| rplM | Rv3443c | 2.16553 | 0.001 |
| rplN | Rv0714 | 3.26035 | 0.00005 |
| rplO | Rv0723 | -2.3141 | 0.00005 |
| rplP | Rv0708 | -2.39926 | 0.00895 |
| rplQ | Rv3456c | -2.91494 | 0.00005 |
| rplS | Rv2904c | 3.19991 | 0.00005 |
| rplT | Rv1643 | 3.57238 | 0.00005 |
| rplU | Rv2442c | 2.29538 | 0.00005 |
| rplX | Rv0715 | 2.31685 | 0.03935 |
| rplY | Rv1015c | 3.66495 | 0.00005 |
| rpmA | Rv2441c | 2.79415 | 0.00005 |
| rpmB2 | Rv2058c | 2.59772 | 0.00675 |
| rpmE | Rv1298 | 2.33886 | 0.00005 |
| rpmF | Rv0979A | 2.41464 | 0.00005 |
| rpmI | Rv1642 | 3.44763 | 0.00005 |
| rpoC | Rv0668 | -3.38857 | 0.00005 |
| rpoZ | Rv1390 | 3.06101 | 0.00005 |
| rpsB | Rv2890c | 2.2449 | 0.00005 |
| rpsC | Rv0707 | -2.2829 | 0.00345 |
| rpsF | Rv0053 | 3.72979 | 0.00005 |
| rpsJ | Rv0700 | 3.45303 | 0.00005 |
| rpsO | Rv2785c | 3.59029 | 0.00005 |
| rpsP | Rv2909c | 2.29628 | 0.00005 |
| rpsQ | Rv0710 | -3.06302 | 0.00005 |
| rpsR1 | Rv0055 | 2.93883 | 0.00005 |
| rpsT | Rv2412 | 2.26741 | 0.00005 |
| sigE | Rv1221 | 2.4162 | 0.00005 |
| ssb | Rv0054 | 3.41497 | 0.00005 |
| trmD | Rv2906c | 2.5583 | 0.00005 |
| tsnR | Rv1644 | 3.68526 | 0.00005 |
| tuf | Rv0685 | -2.19224 | 0.00005 |
| ung | Rv2976c | 2.90636 | 0.00005 |
| rpmG2 | Rv0634B | 3.47612 | 0.00005 |
| **Insertion seqs and phages** |  |  |  |
| Rv0031 | Rv0031 | 3.18432 | 0.00015 |
| Rv0095c | Rv0095c | 4.24805 | 0.00005 |
| Rv1034c | Rv1034c | 2.98256 | 0.0123 |
| Rv1128c | Rv1128c | 2.47344 | 0.00005 |
| Rv1199c | Rv1199c | -Inf | 0.0001 |
| Rv1584c | Rv1584c | 3.19171 | 0.01015 |
| Rv1588c | Rv1588c | 4.21033 | 0.00005 |
| Rv2085 | Rv2085 | -3.38152 | 0.00575 |
| Rv2655c | Rv2655c | 2.61837 | 0.00005 |
| Rv2656c | Rv2656c | 2.50793 | 0.0001 |
| Rv2659c | Rv2659c | 2.419 | 0.00005 |
| Rv2791c | Rv2791c | -2.01875 | 0.0061 |
| Rv2812 | Rv2812 | 2.31619 | 0.0443 |
| Rv2886c | Rv2886c | 2.51873 | 0.0307 |
| Rv3191c | Rv3191c | 2.24322 | 0.00005 |
| Rv3637 | Rv3637 | 2.78032 | 0.00525 |
| Rv3638 | Rv3638 | 2.73498 | 0.00025 |
| Rv3640c | Rv3640c | 2.63766 | 0.0001 |
| Rv3750c | Rv3750c | 2.26379 | 0.00015 |
| Rv3751 | Rv3751 | 2.93831 | 0.0001 |
| **Intermediary metabolism and respiration** |  |  |  |
| aceE | Rv2241 | -2.48713 | 0.00005 |
| ackA | Rv0409 | -2.19659 | 0.03045 |
| adk | Rv0733 | -3.21828 | 0.024 |
| ald | Rv2780 | 2.86652 | 0.00005 |
| aldC | Rv2858c | -3.05527 | 0.0009 |
| argG | Rv1658 | -2.29452 | 0.00005 |
| asd | Rv3708c | -3.19858 | 0.00005 |
| atpA | Rv1308 | -4.01954 | 0.00005 |
| atpC | Rv1311 | -4.24104 | 0.00005 |
| atpD | Rv1310 | -3.54417 | 0.00005 |
| atpE | Rv1305 | -2.55626 | 0.00005 |
| atpF | Rv1306 | -3.17556 | 0.00005 |
| atpG | Rv1309 | -3.35319 | 0.00005 |
| atpH | Rv1307 | -3.38931 | 0.00005 |
| atsB | Rv3299c | -2.00624 | 0.00005 |
| bfrB | Rv3841 | 2.8068 | 0.0001 |
| bglS | Rv0186 | 2.32517 | 0.00005 |
| canA | Rv1284 | 2.85052 | 0.00005 |
| ccdA | Rv0527 | -2.83801 | 0.00005 |
| celA2b | Rv1090 | 2.24317 | 0.00015 |
| citE | Rv2498c | -2.54843 | 0.00915 |
| clpC2 | Rv2667 | 2.01884 | 0.0012 |
| clpP2 | Rv2460c | -2.74241 | 0.00015 |
| cobO | Rv2849c | -2.56466 | 0.00285 |
| cobQ1 | Rv0255c | -2.00542 | 0.00005 |
| csd | Rv1464 | -3.46425 | 0.00005 |
| cyp139 | Rv1666c | 2.36883 | 0.006 |
| cysE | Rv2335 | -2.43861 | 0.00005 |
| cysK2 | Rv0848 | 3.78726 | 0.0052 |
| dhaA | Rv2579 | 2.13133 | 0.00005 |
| dipZ | Rv2874 | 2.65324 | 0.00005 |
| eno | Rv1023 | -2.12428 | 0.00005 |
| fbiB | Rv3262 | -2.51982 | 0.0003 |
| folC | Rv2447c | -2.45561 | 0.0177 |
| folP2 | Rv1207 | -2.31482 | 0.0002 |
| galE2 | Rv0501 | 2.15712 | 0.00005 |
| gcvB | Rv1832 | 2.0115 | 0.00005 |
| gdh | Rv2476c | -3.26277 | 0.00005 |
| ggtA | Rv0773c | -2.38284 | 0.0004 |
| glgP | Rv1328 | -2.29849 | 0.00005 |
| glpQ1 | Rv3842c | 3.05074 | 0.00005 |
| gnd2 | Rv1122 | -2.90711 | 0.00005 |
| hemE | Rv2678c | -2.59516 | 0.0005 |
| hemY | Rv2677c | -2.4453 | 0.00115 |
| hisA | Rv1603 | -2.36974 | 0.00005 |
| hisF | Rv1605 | -2.3385 | 0.00005 |
| ilvC | Rv3001c | -3.65483 | 0.00005 |
| ispF | Rv3581c | 2.03691 | 0.00865 |
| lat | Rv3290c | 4.34531 | 0.00005 |
| leuB | Rv2995c | -2.50186 | 0.00045 |
| lipC | Rv0220 | -2.44001 | 0.00005 |
| lipF | Rv3487c | -3.36813 | 0.00005 |
| lipT | Rv2045c | -2.13535 | 0.00005 |
| lipU | Rv1076 | 2.18014 | 0.00005 |
| lpdC | Rv0462 | -2.03403 | 0.00005 |
| mapA | Rv0734 | -2.67891 | 0.00005 |
| mdh | Rv1240 | -2.92936 | 0.00005 |
| moaB2 | Rv0984 | -2.81626 | 0.0048 |
| moaX | Rv3323c | 2.63671 | 0.0003 |
| moeA1 | Rv0994 | -2.97887 | 0.00005 |
| moeY | Rv1355c | 3.65814 | 0.00005 |
| mscR | Rv2259 | -2.00952 | 0.00005 |
| mtr | Rv2855 | -2.06457 | 0.0001 |
| mycP3 | Rv0291 | -4.1378 | 0.00005 |
| mycP4 | Rv3449 | 3.57856 | 0.00665 |
| nuoD | Rv3148 | -3.41709 | 0.014 |
| nuoF | Rv3150 | -3.40565 | 0.0195 |
| nuoG | Rv3151 | -3.00949 | 0.00045 |
| nuoH | Rv3152 | -2.76609 | 0.0238 |
| nuoJ | Rv3154 | -2.74999 | 0.01065 |
| nuoL | Rv3156 | -3.13453 | 0.0018 |
| nuoM | Rv3157 | -2.95722 | 0.00325 |
| nuoN | Rv3158 | -2.03026 | 0.02575 |
| obg | Rv2440c | 4.08808 | 0.00005 |
| opcA | Rv1446c | -2.66622 | 0.0009 |
| panB | Rv2225 | 2.47054 | 0.00005 |
| pcd | Rv3293 | -2.55928 | 0.00005 |
| pepN | Rv2467 | -2.55443 | 0.00005 |
| pgk | Rv1437 | -2.76511 | 0.00005 |
| plcD | Rv1755c | 2.95493 | 0.00005 |
| pth | Rv1014c | 4.06207 | 0.00005 |
| purL | Rv0803 | -2.60277 | 0.00005 |
| qcrA | Rv2195 | -3.2839 | 0.0021 |
| qcrB | Rv2196 | -2.3278 | 0.00275 |
| rmlC | Rv3465 | -2.09611 | 0.00005 |
| Rv0077c | Rv0077c | 2.12084 | 0.0001 |
| Rv0148 | Rv0148 | -2.23527 | 0.00005 |
| Rv0149 | Rv0149 | -2.57129 | 0.00005 |
| Rv0213c | Rv0213c | 2.21559 | 0.0012 |
| Rv0247c | Rv0247c | -3.52316 | 0.005 |
| Rv0248c | Rv0248c | -2.44407 | 0.00005 |
| Rv0520 | Rv0520 | 2.10551 | 0.0024 |
| Rv0526 | Rv0526 | -2.01554 | 0.03005 |
| Rv0654 | Rv0654 | 2.98576 | 0.00005 |
| Rv0828c | Rv0828c | 2.51396 | 0.0157 |
| Rv0913c | Rv0913c | -2.02773 | 0.0001 |
| Rv0958 | Rv0958 | -2.20806 | 0.0003 |
| Rv1105 | Rv1105 | 2.22001 | 0.0093 |
| Rv1144 | Rv1144 | 3.78366 | 0.00005 |
| Rv1220c | Rv1220c | -2.55614 | 0.00005 |
| Rv1318c | Rv1318c | 4.13101 | 0.00005 |
| Rv1319c | Rv1319c | 3.19527 | 0.00005 |
| Rv1320c | Rv1320c | 2.21184 | 0.00005 |
| Rv1403c | Rv1403c | 2.23748 | 0.0001 |
| Rv1405c | Rv1405c | 4.33311 | 0.00005 |
| Rv1465 | Rv1465 | -3.73305 | 0.01065 |
| Rv1524 | Rv1524 | -2.2282 | 0.0001 |
| Rv1703c | Rv1703c | -2.52159 | 0.00005 |
| Rv1751 | Rv1751 | -3.24607 | 0.00005 |
| Rv1771 | Rv1771 | -2.00638 | 0.0102 |
| Rv1833c | Rv1833c | 2.27918 | 0.00005 |
| Rv1882c | Rv1882c | 2.78191 | 0.00005 |
| Rv1990A | Rv1990A | 5.22046 | 0.00005 |
| Rv2036 | Rv2036 | 3.6074 | 0.01265 |
| Rv2739c | Rv2739c | -2.24132 | 0.00005 |
| Rv2850c | Rv2850c | -2.38282 | 0.0001 |
| Rv2951c | Rv2951c | -2.39328 | 0.00005 |
| Rv3032 | Rv3032 | -2.2129 | 0.00005 |
| Rv3137 | Rv3137 | -3.35613 | 0.00005 |
| Rv3342 | Rv3342 | -2.83361 | 0.02375 |
| Rv3401 | Rv3401 | -2.60863 | 0.00005 |
| Rv3406 | Rv3406 | 2.11652 | 0.0002 |
| Rv3671c | Rv3671c | -2.17394 | 0.00005 |
| Rv3700c | Rv3700c | -2.12962 | 0.0001 |
| Rv3726 | Rv3726 | -2.3469 | 0.00005 |
| Rv3814c | Rv3814c | -2.30093 | 0.00005 |
| Rv3815c | Rv3815c | -3.06801 | 0.00005 |
| sdhA | Rv3318 | -2.24241 | 0.0008 |
| sdhB | Rv3319 | -2.76575 | 0.00155 |
| serA1 | Rv2996c | -2.48343 | 0.00005 |
| serB2 | Rv3042c | -2.31901 | 0.00005 |
| tal | Rv1448c | -2.83392 | 0.00005 |
| thiC | Rv0423c | -2.06145 | 0.00005 |
| thiD | Rv0422c | -2.09833 | 0.0029 |
| thrC | Rv1295 | -2.65833 | 0.0039 |
| tpi | Rv1438 | -3.02361 | 0.0004 |
| trpA | Rv1613 | -3.39556 | 0.0144 |
| trpB | Rv1612 | -2.09477 | 0.04925 |
| trxB1 | Rv1471 | 2.29872 | 0.00005 |
| udgA | Rv0322 | 2.351 | 0.002 |
| zwf2 | Rv1447c | -3.53842 | 0.0001 |
| ligD | Rv0938 | -2.14301 | 0.01055 |
| glgA | Rv1212c | 2.55326 | 0.00005 |
| gpgP | Rv2419c | -2.57128 | 0.00665 |
| kshA | Rv3526 | 2.17383 | 0.00005 |
| dprE2 | Rv3791 | -3.46553 | 0.00005 |
| **Lipid metabolism** |  |  |  |
| accA1 | Rv2501c | -2.09601 | 0.00005 |
| accD4 | Rv3799c | -2.46602 | 0.04055 |
| accD6 | Rv2247 | -2.10844 | 0.00005 |
| echA1 | Rv0222 | -3.09074 | 0.00005 |
| echA17 | Rv3039c | -2.31291 | 0.00005 |
| echA19 | Rv3516 | 2.12864 | 0.00005 |
| echA8 | Rv1070c | -2.1345 | 0.0015 |
| fadB | Rv0860 | -2.11033 | 0.00005 |
| fadD12 | Rv1427c | -2.00645 | 0.002 |
| fadD14 | Rv1058 | 2.62074 | 0.00005 |
| fadD18 | Rv3513c | 2.8152 | 0.01475 |
| fadD2 | Rv0270 | -2.13305 | 0.00005 |
| fadD22 | Rv2948c | -3.54557 | 0.01155 |
| fadD25 | Rv1521 | -2.16769 | 0.00005 |
| fadE16 | Rv1679 | -2.65494 | 0.00805 |
| fadE21 | Rv2789c | -2.55396 | 0.00005 |
| fadE26 | Rv3504 | 2.36259 | 0.00005 |
| inhA | Rv1484 | -2.71644 | 0.00005 |
| mcr | Rv1143 | 2.66474 | 0.00005 |
| papA1 | Rv3824c | -2.05355 | 0.00005 |
| papA4 | Rv1528c | 2.87068 | 0.0002 |
| pimA | Rv2610c | -2.48243 | 0.00035 |
| pks1 | Rv2946c | -2.94784 | 0.00035 |
| pks11 | Rv1665 | 3.13689 | 0.00005 |
| pks12 | Rv2048c | -2.75442 | 0.00005 |
| pks13 | Rv3800c | -2.70467 | 0.00005 |
| pks2 | Rv3825c | -3.37812 | 0.00005 |
| pks3 | Rv1180 | -3.70559 | 0.00005 |
| pks4 | Rv1181 | -2.27719 | 0.00005 |
| Rv1544 | Rv1544 | -2.85536 | 0.00005 |
| Rv3720 | Rv3720 | -2.62072 | 0.03925 |
| Rv0947c | Rv0947c | 2.15152 | 0.0037 |
| **PE/PPE** |  |  |  |
| lipX | Rv1169c | 3.35563 | 0.00005 |
| PE10 | Rv1089 | 2.69743 | 0.04335 |
| PE16 | Rv1430 | 2.19929 | 0.00005 |
| PE17 | Rv1646 | 2.95625 | 0.00005 |
| PE20 | Rv1806 | 6.205 | 0.0008 |
| PE22 | Rv2107 | 2.28141 | 0.00945 |
| PE32 | Rv3622c | 3.02566 | 0.0015 |
| PE34 | Rv3746c | 2.65819 | 0.00315 |
| PE8 | Rv1040c | 3.72198 | 0.00005 |
| PE9 | Rv1088 | 2.44269 | 0.00355 |
| PE_PGRS18 | Rv0980c | 3.66771 | 0.00005 |
| PE_PGRS29 | Rv1468c | 2.16938 | 0.00005 |
| PPE11 | Rv0453 | 2.37487 | 0.00005 |
| PPE15 | Rv1039c | 2.69734 | 0.00005 |
| PPE18 | Rv1196 | -2.61612 | 0.00005 |
| PPE20 | Rv1387 | -2.54779 | 0.00005 |
| PPE29 | Rv1801 | 3.06812 | 0.00005 |
| PPE31 | Rv1807 | 2.95054 | 0.00005 |
| PPE34 | Rv1917c | 2.06554 | 0.00005 |
| PPE39 | Rv2353c | 3.29059 | 0.0004 |
| PPE4 | Rv0286 | -4.32175 | 0.00005 |
| PPE46 | Rv3018c | 2.4134 | 0.00005 |
| PPE51 | Rv3136 | -2.88526 | 0.00005 |
| PPE60 | Rv3478 | -2.08351 | 0.0001 |
| PPE65 | Rv3621c | 2.03028 | 0.00015 |
| PPE69 | Rv3892c | 2.91507 | 0.00005 |
| **Regulatory proteins** |  |  |  |
| cmtR | Rv1994c | 3.29144 | 0.00005 |
| embR | Rv1267c | 2.56007 | 0.00005 |
| furA | Rv1909c | 4.83829 | 0.00005 |
| lexA | Rv2720 | 3.67785 | 0.00005 |
| pknH | Rv1266c | 4.64903 | 0.00005 |
| regX3 | Rv0491 | -2.84607 | 0.0185 |
| Rv0195 | Rv0195 | 2.3409 | 0.0022 |
| Rv0260c | Rv0260c | 2.07904 | 0.02365 |
| Rv0474 | Rv0474 | 2.44321 | 0.00005 |
| Rv0576 | Rv0576 | 4.0975 | 0.00005 |
| Rv0653c | Rv0653c | 2.09552 | 0.00795 |
| Rv0744c | Rv0744c | 2.09386 | 0.00025 |
| Rv1129c | Rv1129c | 4.23415 | 0.00005 |
| Rv1219c | Rv1219c | 2.10052 | 0.0441 |
| Rv1773c | Rv1773c | 2.05996 | 0.00005 |
| Rv1990c | Rv1990c | 6.44758 | 0.00005 |
| Rv2021c | Rv2021c | 3.35056 | 0.00005 |
| Rv2034 | Rv2034 | 4.74035 | 0.00215 |
| Rv2640c | Rv2640c | 2.78705 | 0.00005 |
| Rv2642 | Rv2642 | 3.17723 | 0.002 |
| Rv2884 | Rv2884 | 2.18238 | 0.00005 |
| Rv2887 | Rv2887 | 2.28477 | 0.0384 |
| Rv3060c | Rv3060c | 2.44661 | 0.00005 |
| Rv3066 | Rv3066 | 2.27797 | 0.0005 |
| Rv3183 | Rv3183 | 5.84122 | 0.00045 |
| Rv3334 | Rv3334 | 3.93125 | 0.00005 |
| Rv3405c | Rv3405c | 2.43497 | 0.00005 |
| Rv3736 | Rv3736 | 2.18193 | 0.00005 |
| Rv3833 | Rv3833 | 2.32519 | 0.01095 |
| Rv3840 | Rv3840 | 2.57747 | 0.00075 |
| whiA | Rv1423 | -2.49615 | 0.0264 |
| whiB6 | Rv3862c | 4.57979 | 0.00005 |
| whiB7 | Rv3197A | 3.55957 | 0.00005 |
| mce2R | Rv0586 | 3.05025 | 0.00005 |
| clgR | Rv2745c | -2.42141 | 0.00005 |
| moaR1 | Rv3124 | 2.22521 | 0.00025 |
| **Virulence,detoxification and Adaptation** |  |  |  |
| bpoB | Rv1123c | -2.43137 | 0.00085 |
| cfp29 | Rv0798c | -2.38631 | 0.0117 |
| groEL1 | Rv3417c | -2.96451 | 0.00005 |
| groEL2 | Rv0440 | -3.11798 | 0.00005 |
| groES | Rv3418c | -3.25264 | 0.00005 |
| mce2F | Rv0594 | 3.23965 | 0.00005 |
| mce3D | Rv1969 | 2.4991 | 0.04915 |
| proX | Rv3759c | -2.26053 | 0.00005 |
| Rv0024 | Rv0024 | -2.1975 | 0.01655 |
| Rv1566c | Rv1566c | 2.52397 | 0.00005 |
| Rv3269 | Rv3269 | 4.8525 | 0.00005 |
| Rv3660c | Rv3660c | 3.84231 | 0.00005 |
| treZ | Rv1562c | 2.01048 | 0.0044 |
| vapC25 | Rv0277c | 2.73446 | 0.00005 |
| vapC4 | Rv0595c | 4.43352 | 0.00005 |
| vapB4 | Rv0596c | 4.72428 | 0.00005 |
| vapB28 | Rv0608 | 2.01383 | 0.0022 |
| vapC28 | Rv0609 | 2.33262 | 0.0009 |
| vapB30 | Rv0623 | 2.08008 | 0.0003 |
| vapC30 | Rv0624 | 2.57974 | 0.0322 |
| vapB5 | Rv0626 | 3.24972 | 0.00005 |
| vapC5 | Rv0627 | 3.31054 | 0.00005 |
| vapB8 | Rv0664 | -2.67393 | 0.0058 |
| vapC8 | Rv0665 | -2.35108 | 0.0468 |
| mazF3 | Rv1102c | 3.28863 | 0.0073 |
| vapB33 | Rv1241 | -2.66075 | 0.0002 |
| vapB10 | Rv1398c | 2.92267 | 0.00005 |
| vapC11 | Rv1561 | 10.2802 | 0.00005 |
| vapC12 | Rv1720c | 2.432 | 0.02835 |
| vapC13 | Rv1838c | 2.3121 | 0.01025 |
| higA | Rv1956 | 4.28549 | 0.00005 |
| parE1 | Rv1959c | 3.19892 | 0.0021 |
| parD1 | Rv1960c | 2.74515 | 0.00905 |
| vapB36 | Rv1982A | 2.93924 | 0.00005 |
| mazE6 | Rv1991A | 4.57803 | 0.00005 |
| vapB15 | Rv2009 | 3.75368 | 0.00005 |
| vapC15 | Rv2010 | 3.93763 | 0.00005 |
| vapB37 | Rv2104c | 2.07336 | 0.0001 |
| parD2 | Rv2142A | 3.0728 | 0.0004 |
| parE2 | Rv2142c | 3.23526 | 0.0142 |
| mazF8 | Rv2274c | 3.25381 | 0.00005 |
| vapC18 | Rv2546 | 3.16338 | 0.00105 |
| vapB41 | Rv2601A | 2.54337 | 0.00115 |
| vapB46 | Rv3385c | 2.40991 | 0.00005 |
| **Unknown** |  |  |  |
| mcr11 | EBG00000313343 | 3.8735 | 0.00005 |
| rrf | EBG00000313349 | 5.0547 | 0.00005 |
| mcr3 | EBG00000313377 | 3.33917 | 0.0417 |
| mcr7 | EBG00000313380 | -2.68369 | 0.0002 |
| Rv0061c | Rv0061c | 2.22052 | 0.00005 |
| mymT | Rv0186A | 4.35331 | 0.00005 |
| icl1 | Rv0467 | 2.91587 | 0.00005 |
| oxyR'-1 | Rv2427A | 2.06699 | 0.00585 |
| bkdC | Rv2495c | -2.87856 | 0.0001 |
| bkdB | Rv2496c | -2.14517 | 0.00145 |
| Rv3098A | Rv3098A | 2.98035 | 0.00435 |
| Rv3136A | Rv3136A | -2.89266 | 0.00005 |
| Rv3190A | Rv3190A | 2.56697 | 0.00005 |
| Rv1046c | Rv1046c | 2.10468 | 0.00005 |
| Rv2645 | Rv2645 | 2.21647 | 0.00345 |
