# Supplementary material for: Structural, functional and biological insights into the role of Mycobacterium tuberculosis VapBC11 toxin–antitoxin system: targeting a tRNase to tackle mycobacterial adaptation
Source: Nucleic Acids Res. 2018 Oct 17;46(21):11639–55. doi: 10.1093/nar/gky924 (PMC6265470; doi:10.1093/nar/gky924)
Supplement: Supplementary Data [file gky924_supplemental_files.zip › Table_S1_VapBC11.docx]

**Supplementary Table S1:**

| **S.No.** | **PRIMER NAME** | **NUCLEOTIDE SEQUENCE** |
| --- | --- | --- |
| 1 | VapC11_Wt_NheI_NdeI_Fwd | ATGCCATATGGCTAGCATCCTCATCGCCACATCGGCCTG |
| 2 | VapC11_D5A_NheI_NdeI_Fwd | ATGCCATATGGCTAGCATCCTCATCGCCACATCGGCCTG |
| 3 | VapC11_HindIII_Xho_Rev | CGTACTCGAGAAGCTTCACCGAAACGATGCGGC |
| 4 | VapB11_NdeI_Fwd | ATGCACATATGAGTCGCACCAACATCGACATC |
| 5 | VapB11_XhoI_Rev | ACTCACTCGAGTCAGTCTGGGCGATCGCTTCGC |
| 6 | VapC11_R14E_Fwd | GTGGAGTACTTCGAGGCCACCGGATCAATCG |
| 7 | VapC11_R87E_Fwd | CTATCGCGCCGCCGAGCGCGCCGGCGAAACGG |
| 8 | VapC11_R94E_Fwd | CGGCGAAACGGTTGAGAGCATCAACGACTGC |
| 9 | LeuT_150bp_Up_NheI_Fwd | ATGCGCTAGCCGACTCGCCTGTATGTCGTG |
| 10 | LeuT_150bp_down_XhoI_Rev | ATGCACTCGAGGCGACCGAGCTGAACC |
| 11 | LeuT_T7Pro_Fwd | TAATACGACTCACTATAGGGGCGAGTGGCGGAATG |
| 12 | LeuT_Rev | TGGGCGAAGGGGGACTTG |
| 13 | VapBC11_800bp_Up_Fwd | GGGAGGCCTGGTCCCGGTGGGTGGCGGCGGCTGC |
| 14 | VapBC11_800bp_Up_Rev | GGGACTAGTGGTGCGCGACATACACCAACGATA |
| 15 | VapBC11_800bp_Down_Fwd | GGGCCATGGACCAACCTGCAGGCCGCATCGTTTCGG |
| 16 | VapBC11_800bp_Down_Rev | GGGACTAGTCGGCTCGCTGGCCACTCTCGCG |
| 17 | HR_Primer Fwd | TTGGAACCGGGGTCGCCGGCTTACC |
| 18 | HR_Primer Rev | GTCGCCGATTATCGGCGATAATTCC |
| 19 | Rv1560_qPCR_Fwd | GGGGGAATTCCATATGGTGTATCGTTGGTGTATGTCGCG |
| 20 | Rv1560_qPCR_Rev | GGGAAGCTTTCAGTCTGGGCGATCGCTTCGCAAG |
| 21 | Rv1561_qPCR_Fwd | CGCTGTCGAAGTACGCCGGCTGCTG |
| 22 | Rv1561_qPCR_Rev | CGCCGGGCGGCGCGATAGAT |
| 23 | Rv2660c_qPCR_Fwd | GCAGCAACAGGCCAGGCTAGCCAG |
| 24 | Rv2660c_qPCR_Rev | CCCAGTATCGCGCACCACGATTGAC |
| 25 | Rv2661c_qPCR_Fwd | GGTGGCCCGTAGTGCGCGTCGAGC |
| 26 | Rv2661c_qPCR_Rev | CCGCACCCGCGCAACCGAGC |
| 27 | Rv2662_qPCR_Fwd | TGCGGGACTTCACAGACTGG |
| 28 | Rv2662_qPCR_Rev | ACCAGCGCAAACAGAAGAGT |
| 29 | Rv2663_qPCR_Fwd | CCGCAAGCACGGCATCAACGACGA |
| 30 | Rv2663_qPCR_Rev | CGTAGAACTTCGGGCGTAGT |
| 31 | Rv2745c_qPCR_Fwd | GAGGTGTCCGATTCGGCGCGGGTG |
| 32 | Rv2745c_qPCR_Rev | CTTGGTGCTGGCGTCAATGGTGGCG |
| 33 | Rv0936_qPCR_Fwd | GGCGGCGGCTTGCGCAACGCC |
| 34 | Rv0936_qPCR_Rev | GGGCCAAATAGCCGACGTAGC |
| 35 | Rv0491_qPCR_Fwd | GTGGAGGACGAGGAGTCGCTGGCCG |
| 36 | Rv0491_qPCR_Rev | TCCCGGGCGGTCACCATGATCACC |
| 37 | Rv1310_qPCR_Fwd | GGTCTGAAGGTGGTCGACCTG |
| 38 | Rv1310_qPCR_Rev | CGTTGGCTTCGGCAAGCTCGAC |
| 39 | Rv3151_qPCR_Fwd | GGTCGCACGCAATGGCGGTGG |
| 40 | Rv3151_qPCR_Rev | GGCGATGCGGGCCGCCAGGAAG |
| 41 | SigA_qPCR_Fwd | ACGAAGACCACGAAGACCTCGAA |
| 42 | SigA_qPCR_Rev | GTAGGCGCGAACCGAGTCGGCGG |
| 43 | SigE_qPCR_Fwd | GGGCACCGCCGTATTCGACGCC |
| 44 | SigE_qPCR_Rev | CCGGCGGCGGACCATGTCCAGG |
| 45 | Rv3290c_qPCR_Fwd | GTGGCGATGGCCCGCTTCGTCGAGA |
| 46 | Rv3290c_qPCR_Rev | AATGCCCCGCGCAGGTGGAGCAC |
