# Supplementary material for: Structural, functional and biological insights into the role of Mycobacterium tuberculosis VapBC11 toxin–antitoxin system: targeting a tRNase to tackle mycobacterial adaptation
Source: Nucleic Acids Res. 2018 Oct 17;46(21):11639–55. doi: 10.1093/nar/gky924 (PMC6265470; doi:10.1093/nar/gky924)

Supplementary Figure S1. Effect of VapC11 overexpression on cell viability and RNA-Seq data validation of DEGs by qPCR. **(A)** Live-dead staining of *M. bovis BCG* harbouring either vector or pTetR-*vapC11* was performed using SYTO-9/propidium. The stained cells were viewed by confocal microscopy at day 0, 1, and 2 post-Atc induction. Scale bar size: 5 μm. **(B)** For RNA-seq data validation, extraction of RNA, cDNA synthesis and qPCR using gene-specific primers was performed as described in the Materials and Methods section. The relative gene expressions were quantified after normalization with *sigA* expression. The data shown is the mean ± standard error obtained from three independent experiments. The values in the parentheses correspond to the fold change observed in the RNA-seq data. ND corresponds to the non-differentially expressed genes.

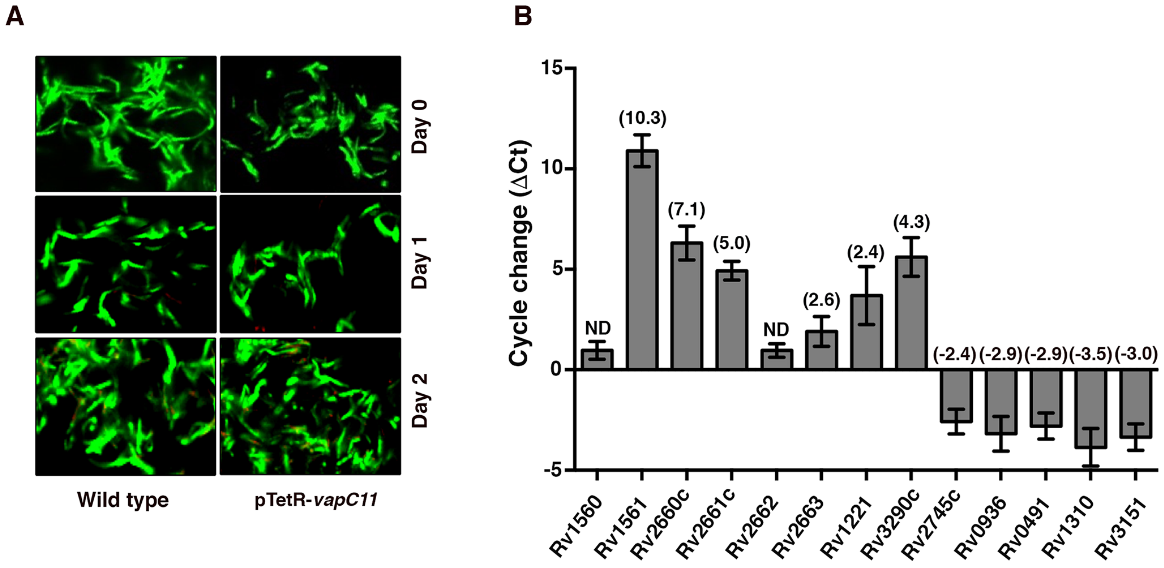

Supplementary Figure S2. VapBC11 locus disruption strategy and validation of locus deletion. **(A)** The *vapBC11* locus was replaced with hygromycin *(hyg)* resistance gene in the genome of *Mtb* by homologous recombination using temperature-sensitive mycobacteriophages. **(B, C)** The construction of the knockout strain was confirmed by Southern blot **(B)** and PCR **(C)**.

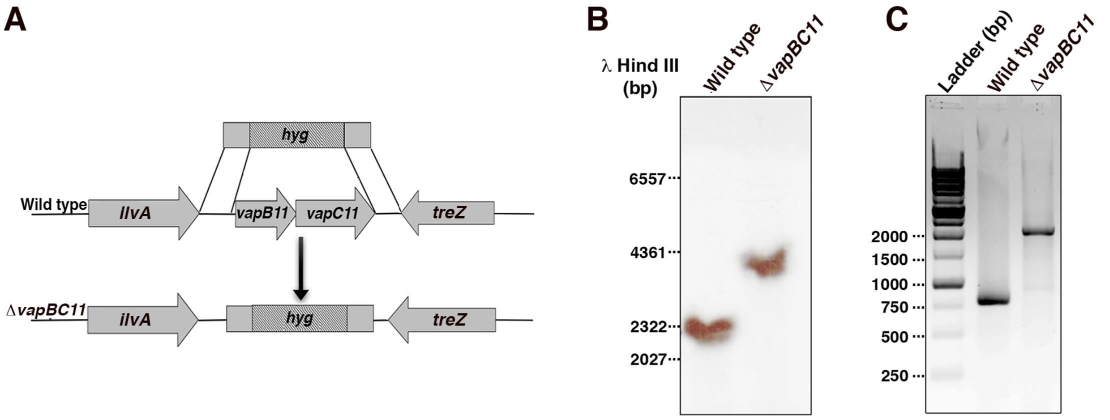

Supplementary Figure S3. The effect of disruption of *vapBC11* locus on survival of *Mtb* upon exposure to different stress conditions. **(A)** For oxidative stress, early log-phase cultures were washed and exposed to 5 mM H_2O_2 for 24 hrs. **(B)** For nitrosative stress, cultures were exposed to 5 mM $NaNO_2$ for 3 days. **(C)** For nutritional stress, early log-phase cultures were washed and resuspended in 1x TBST for 7 days. **(D)** For macrophage experiments, THP-1 cells were infected with various strains at an MOI of 1:10, and colony forming units (CFU) were determined at the designated time points. **(E)** For nonreplicating persisters, the Wayne model of NRP was used, and bacteria were harvested at designated time points. **(F)** The survival of both wild-type and mutant strain was compared upon exposure to drugs including rifampicin (Rif), levofloxacin (Levo) and isoniazid (INH) for 7 days. Bacterial enumerations were performed by dilution plating, followed by incubation for 3-4 weeks. The data presented in these panels is the average of 3 independent experiments. Significant differences were observed for the indicated groups (paired [two-tailed] t-test, *represents p-value <0.05).

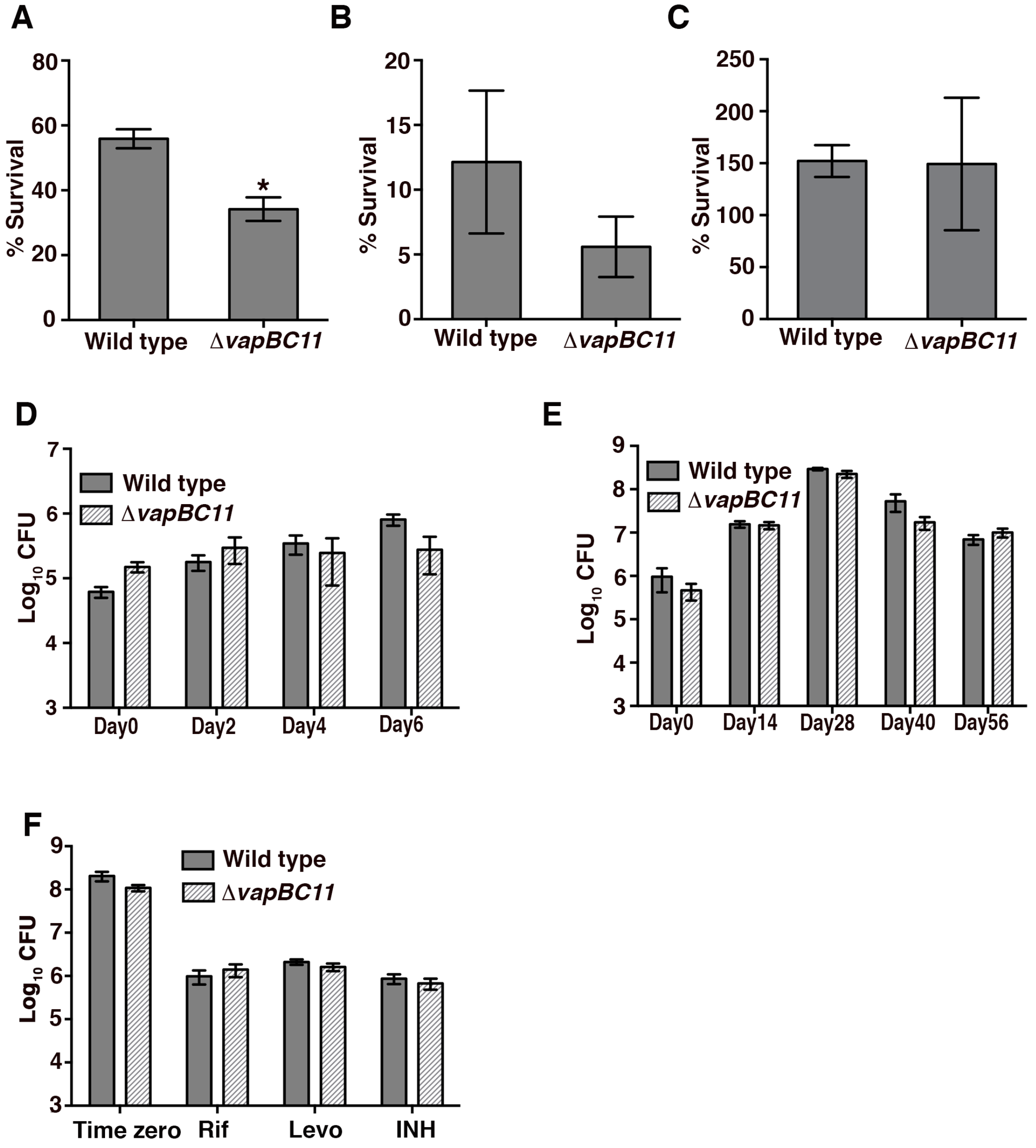

Supplementary Figure S4. Structural superposition of VapBC11 complex with FitAB-DNA complex. The VapBC11 complex structure is shown in surface representation. Toxin molecules are in light grey colour, while the antitoxin molecules are shown in magenta colour. The FitAB-DNA complex structure is shown as surface and cartoon representation. The toxin and antitoxin molecules are shown in light pink and green colours, respectively. A superimposed model showing the RHH DNA-binding domain of VapB11 positioned at a distance comparable to the DNA-binding domain of FitA antitoxins.

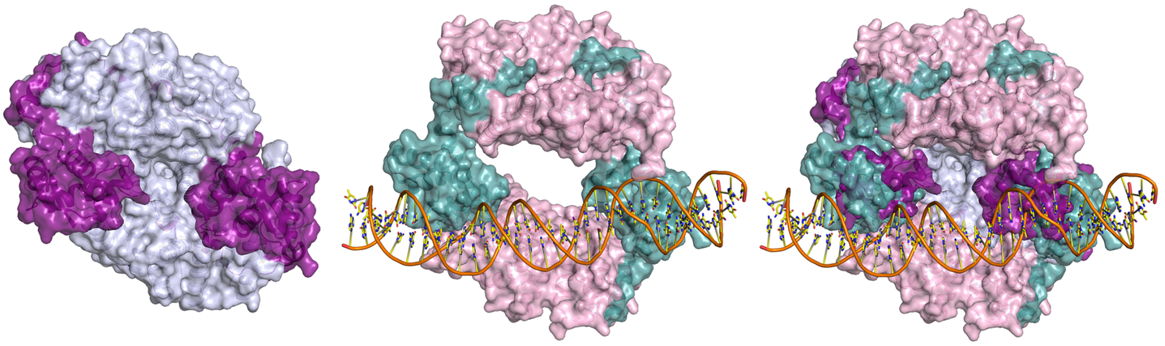

VapBC11 FitAB:DNA Merged-FitAB:DNA:VapBC11

Supplementary Figure S5. Multiple sequence alignment (MSA) of VapC homologs. This panel shows MSAs among VapC homologs sharing identities of 15 to 90 % among themselves. The black asterisk (*) represents the PIN-domain residues. The red asterisk (*) represents the functionally important Arg residue.

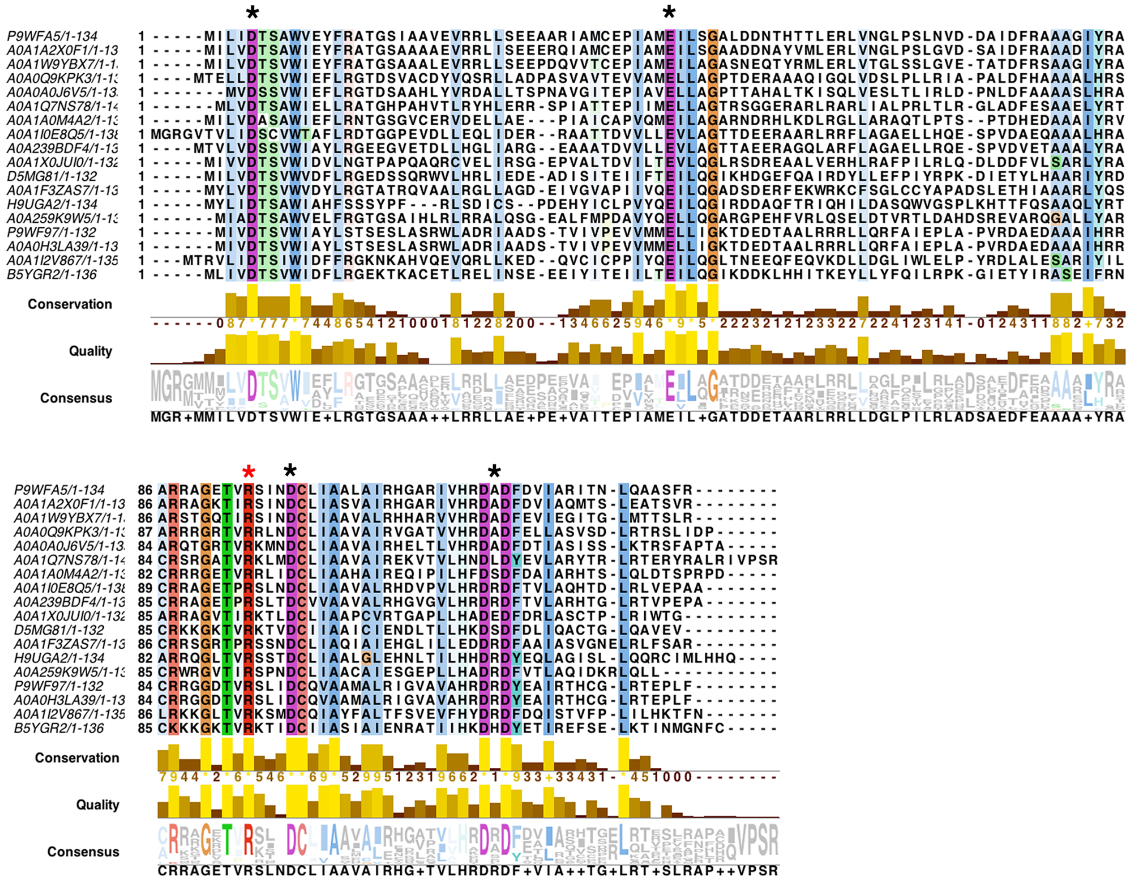

Supplementary Figure S6. Structural superposition of VapC11 homologs. **(A)** The panel shows structural superimposition of the closest VapC11 structural homologs. Homodimeric VapC15 and VapC21 are aligned on the chain A of VapC11 revealing the subtle differences in the relative orientations of the monomers in the homodimers. The common secondary structure features among all VapCs are shown with black labels. VapC21 possesses an additional α-helix, α6, and the secondary structural elements (α6, α7 and α8) are labelled in red. The differences are presented in dotted circles. **(B)** Differences in the overall charge distribution are shown in electrostatic potential representation. The vacuum electrostatic maps were generated using PyMOL with default parameters. Red, blue, and white colours indicate negative, positive and uncharged regions, respectively.

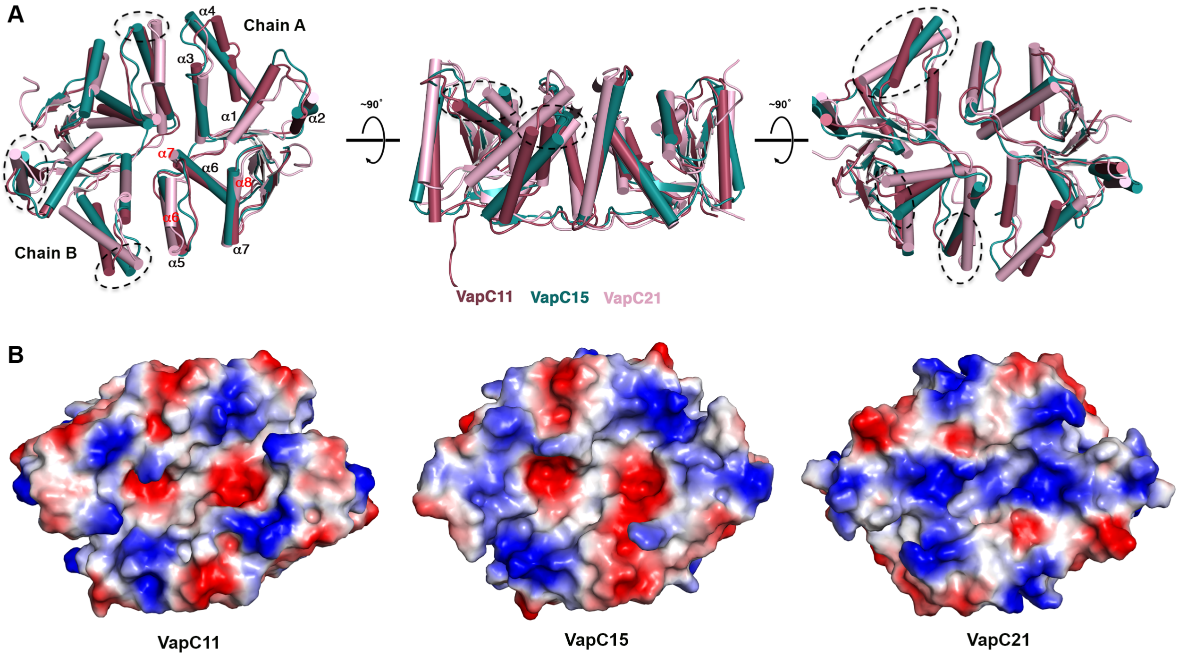

Supplementary Figure S7. Similarities and differences in the structural features in VapBC structural homologs. **(A)** VapBC complex structures showing toxin: antitoxin binding stoichiometry. Monomers of VapC toxin are shown in light blue and dark blue colours. Antitoxin monomers are shown in green and orange colours. VapBC complex structures display major differences at their interaction interfaces. Significant differences are also observed at the VapC: VapC interaction interface. **(B)** Extracted antitoxin (VapB) structures from various VapBC complex structures showing variations in their overall secondary structures. N and C represent crystallographically resolved N- and C-terminal regions. Stoichiometry in parenthesis indicates the mode of active site neutralization by antitoxin.

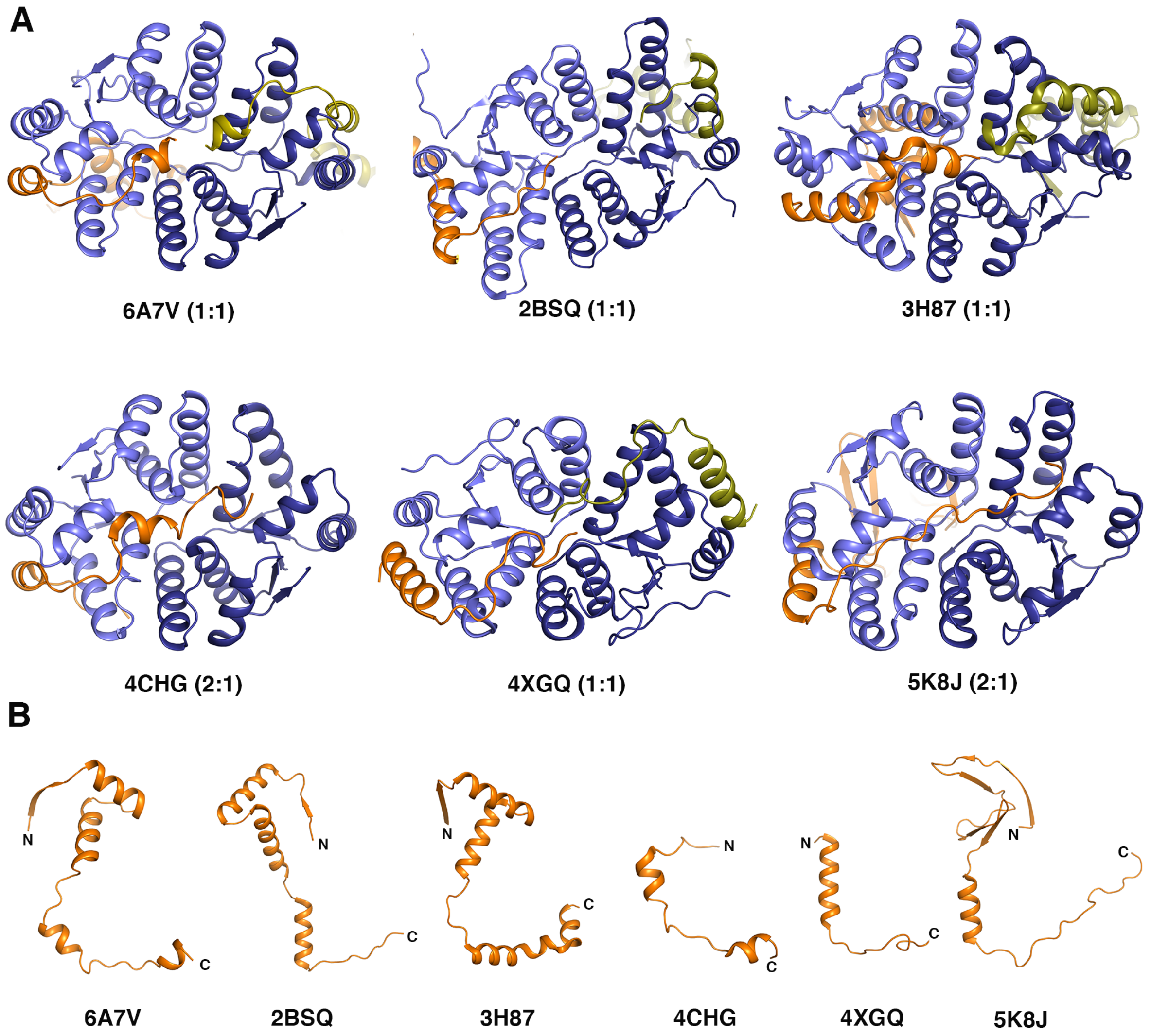

Supplementary Figure S8. Homotetrameric oligomeric state of VapC11. In solution data suggest that VapC11 forms a concentration-dependent homotetramer in solution. The crystal structure reveals that two homodimeric VapC11 molecules interact to form a homotetrameric species. This complex is stabilized via salt bridge and hydrogen bond interactions. The α4 and α5 of the diagonally positioned VapC11 chains participated in these interactions. Each chain is coloured differently, and the interacting residues are shown in ball and stick representation.

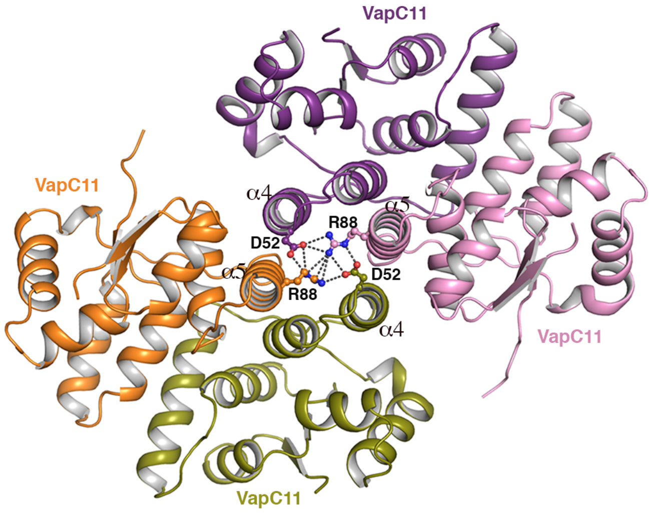

Supplementary Figure S9. MSA of VapB11 homologs and CD spectroscopy analysis of VapC11 mutants. **(A)** Panel showing MSA among VapB homologs that share sequence identity of between ~20 to 90 %. The consensus among VapB proteins is shown as a bar diagram and is coloured distinctly based on conservation. **(B)** The far-UV CD profile shows no secondary structural differences in VapC11^{D5A} and VapC11^{R94E}. CD experiments were performed at 10 μM protein concentrations using a Jasco J-815 CD instrument at 25 °C. **(C)** Affinity measurement of VapC11^{R94E,D5A} mutant with varying concentration of tRNA-Leu^{CAG}. Proteins were biotinylated with an EZ-Link® NHS-PEG$_4$-Biotin, No-Weigh^{TM} format kit (Thermo Scientific), as per the manufacturer's protocol and were desalted using desalting Micro Bio-spin® 6 columns (Bio-Ras). The BLI experiments were performed using high-precision streptavidin (SAX) biosensors (ForteBio, Pall Corporation). The curves observed in the negative scales are probably due to the positive drift observed in the reference sensor. A representative VapC11^{D5A} /tRNA-Leu^{CAG} binding profile, used as a positive control in these experiments, are shown in black. The data fitting is shown in red. The affinity observed for the VapC11^{D5A} /tRNA-Leu^{CAG} complex using SAX and CM5 biosensors (as shown in Figure 5A) was comparable.

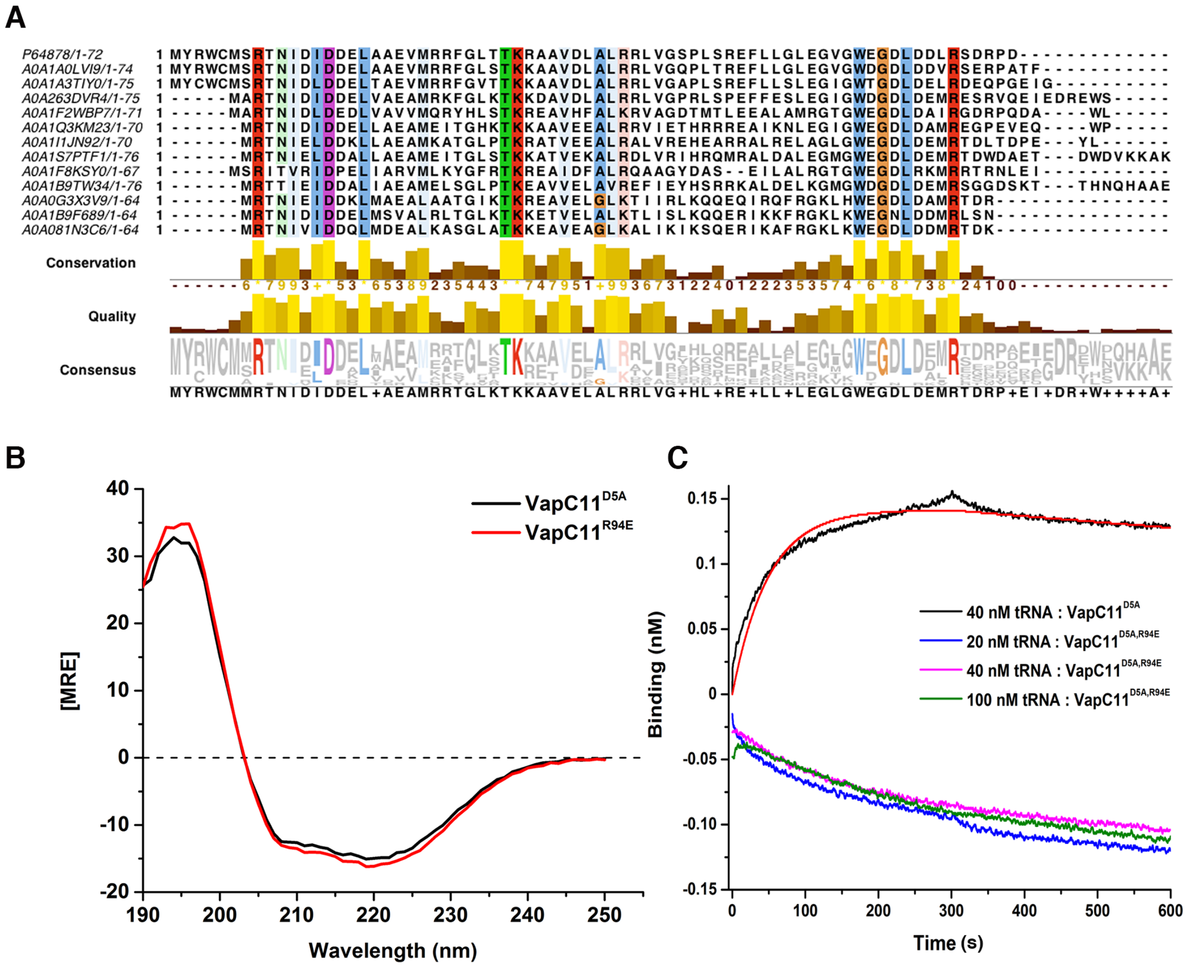

Supplement: Supplementary Data [file gky924_supplemental_files.zip › VapBC11_Supplementary_Material.pdf]
